# Click-Chemistry Cross-Linking of Hyaluronan Graft Copolymers

**DOI:** 10.3390/pharmaceutics14051041

**Published:** 2022-05-11

**Authors:** Mario Saletti, Marco Paolino, Lavinia Ballerini, Germano Giuliani, Gemma Leone, Stefania Lamponi, Marco Andreassi, Claudia Bonechi, Alessandro Donati, Daniele Piovani, Alberto Giacometti Schieroni, Agnese Magnani, Andrea Cappelli

**Affiliations:** 1 Dipartimento di Biotecnologie, Chimica e Farmacia (Dipartimento di Eccellenza 2018–2022), Università degli Studi di Siena, Via Aldo Moro 2, 53100 Siena, Italy; mario.saletti@student.unisi.it (M.S.); lavinia.ballerini@student.unisi.it (L.B.); giuliani5@unisi.it (G.G.); gemma.leone@unisi.it (G.L.); stefania.lamponi@unisi.it (S.L.); marco.andreassi@unisi.it (M.A.); claudia.bonechi@unisi.it (C.B.); alessandro.donati@unisi.it (A.D.); agnese.magnani@unisi.it (A.M.); 2 Istituto di Scienze e Tecnologie Chimiche “G. Natta”-SCITEC (CNR), Via A. Corti 12, 20133 Milano, Italy; piovani@ismac.cnr.it (D.P.); alberto.giacometti@scitec.cnr.it (A.G.S.)

**Keywords:** hyaluronic acid, ferulic acid, click-chemistry, crosslinking, hydrogel

## Abstract

An easy and viable crosslinking procedure by click-chemistry (click-crosslinking) of hyaluronic acid (**HA**) was developed. In particular, the clickable propargyl groups of hyaluronane-based **HA**-**FA**-**Pg** graft copolymers showing low and medium molecular weight values were exploited in crosslinking by click-chemistry by using a hexa(ethylene glycol) spacer. The resulting **HA-FA-HEG-CL** materials showed an apparent lack of in vitro cytotoxic effects, tuneable water affinity, and rheological properties according to the crosslinking degree that suggests their applicability in different biomedical fields.

## 1. Introduction

Hyaluronic acid (**HA**, hyaluronan) is a glycosaminoglycan derivative playing a wide range of important roles in the human body [1]. Among them, **HA** is one of the extracellular matrix (ECM) components, whose main physiological functions range from the maintenance of cell osmotic balance and tissues structural support to lubrication, wear, and the damping of excessive loads on the joints [2]. One of the most intriguing **HA** functions is the constitution of a pericellular coat, which was suggested to affect the early stages of cell adhesion by interacting with the CD44 receptor [3]. The resulting activation of **HA**-CD44 signaling pathways controls different cell biological functions such as angiogenesis, cell migration, proliferation, aggregation, and adhesion to ECM components [2]. Furthermore, thanks to its ability to absorb large amounts of water, **HA** regulates tissues hydratation and represents a lubricant supplementation for the treatment of osteoarticular and eye diseases [4]. **HA** (cross-linked and non-cross-linked) derivatives have been largely used in the formation of biocompatible hydrogels for pharmaceutical and medical applications [5,6,7].

Ferulic acid (**FA)** is a cinnamic acid derivative (i.e., 4-hydroxy-3-methoxycinnamic acid), which is basically allocated in the plant cellular wall [8]. **FA** residues are inserted by means of an ester bond to primary alcohol of arabinose side chains in the cell wall arabinoxylan polysaccharides, where it is then involved in cross-linking polysaccharides and proteins during cell wall synthesis [8,9,10,11,12,13].

**Figure 1:**
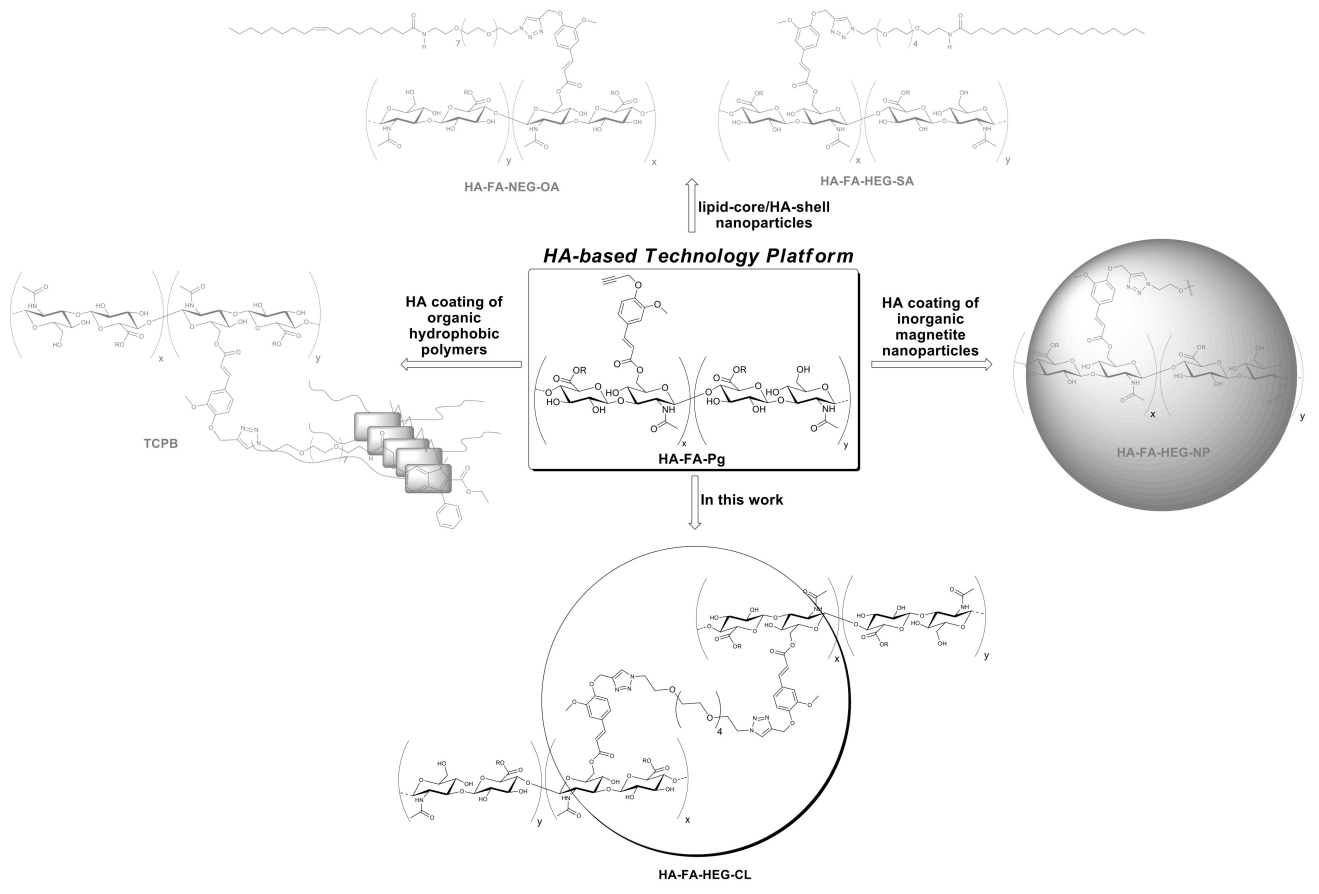
Development and applications of the **HA**-based technology platform employing **HA-FA-Pg** graft copolymers.

In our laboratories, the **HA** macromolecules were grafted with **FA** residues to obtain **HA-FA** graft copolymers [14,15], and subsequently the chemistry was used in the development of a tri-component polymer brush based on a polybenzofulvene derivative bearing nona(ethylene glycol) side chains [16,17]. In particular, this polybenzofulvene cylindrical brush was prepared by means of a convergent approach employing a copper(I)-catalyzed azide-alkyne 1,3-dipolar cycloaddition (CuAAC) of the suitable hyaluronan derivative **HA-FA-Pg** (Figure 1) bearing propargyl groups bound to an **HA** backbone through **FA** fluorophores [16,18]. 

The procedure of coating a polybenzofulvene cylindrical brush surface with **HA** by means of **HA-FA-Pg** graft copolymers was gradually transformed into a technology platform which was applied to the coating of the surfaces of different nanostructures such as liposomes [19], self-assembling micelles [20], and magnetic nanoparticles [21]. 

**Figure 2:**
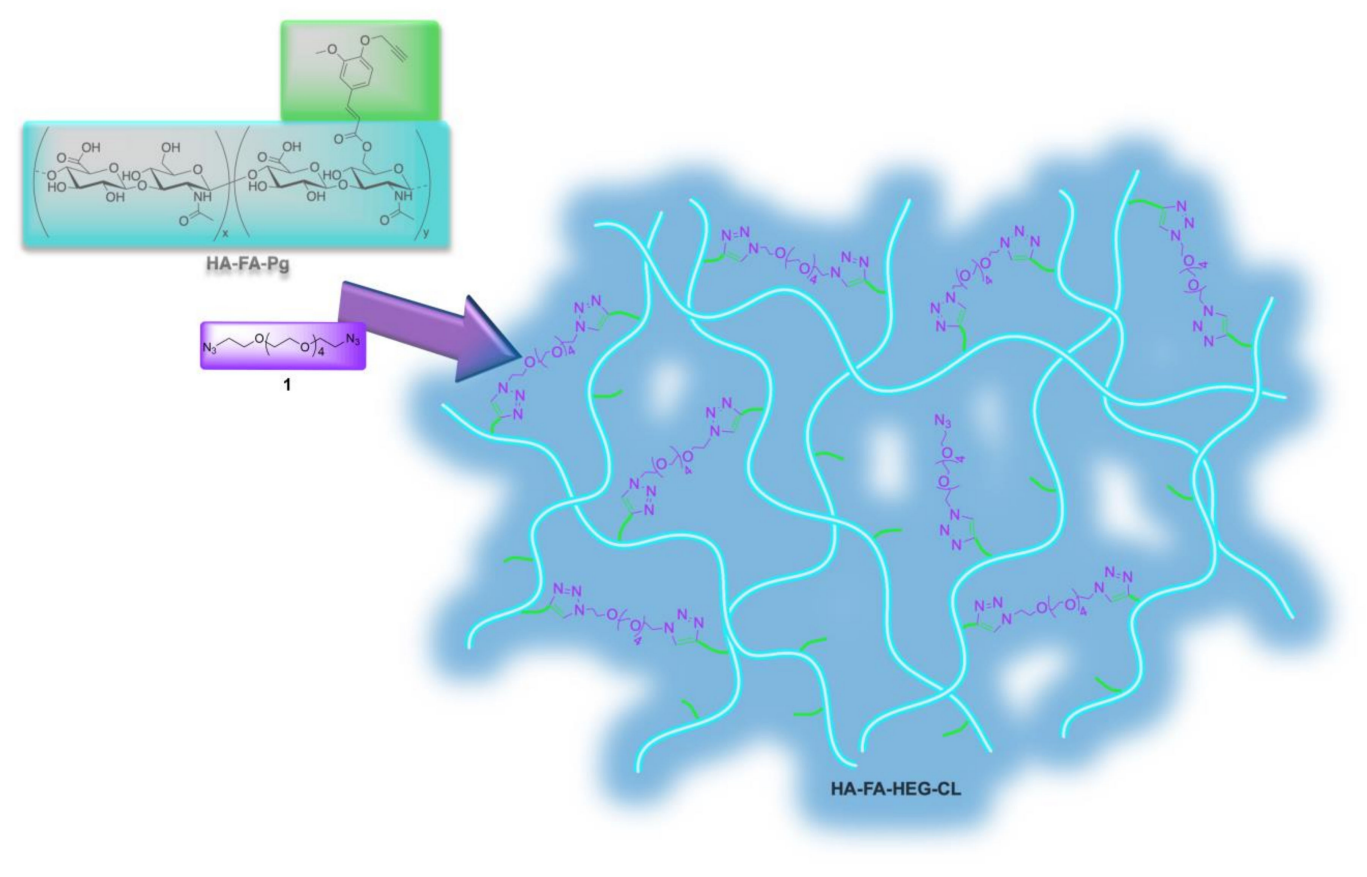
Click-chemistry cross-linking of **HA-FA-Pg** graft copolymers.

With the aim of further extending the application of **HA-FA-Pg** graft copolymers to the development of new materials based on two natural compounds from biorenewable resources, we developed a cross-linking procedure in which the propargyl groups of **HA**-**FA**-**Pg** derivatives showing different molar mass and grafting degree values were exploited in the CuAAC coupling with a hexa(ethylene glycol) derivative terminated with azide groups at both ends (compound **1**, azido-**HEG-**azido) to obtain cross-linked **HA** derivatives (i.e., **HA-FA-HEG-CL**) (Figure 2). 

First, the grafting procedure based on imidazolide derivative **2** was applied to **HA** showing a medium molar mass value (i.e., Mw = 270 KDa) to expand the **HA-FA-Pg** armamentarium in our laboratories. The resulting **HA(270)-FA-Pg** derivatives showing different grafting degree values (i.e., 10, 20, and 40%) were characterized and then used in the CuAAC coupling with compound **1** (azido-**HEG-**azido) to obtain cross-linked **HA** derivatives (i.e., **HA-FA-HEG-CL**) showing different cross-linking densities. The same cross-linking procedure was also applied to the previously published **HA-FA-Pg** graft copolymer showing a low molar mass value (i.e., Mw = 8.7 KDa) and a grafting degree of 20% (i.e., **HA-FA-Pg-3F**) to obtain cross-linked **HA** derivative **HA(8.7)-FA-HEG-CL-20**. The cross-linked **HA** derivatives were characterized from the point of view of their rheological and cytocompatibility features and proposed as biocompatible material with high potentiality in biomedical applications.

## 2. Experimental Section

### 2.1. Synthesis and Characterization

All reagents and solvents were purchased from Sigma–Aldrich and were used as received, with the exceptions noted. Merck TLC aluminum sheets, silica gel 60 F_254_ were used for TLC. NMR spectra were recorded with either a Bruker AMX-600 AVANCE or a Varian Mercury 300 spectrometer in the indicated solvents. The chemical shifts are referenced to the solvent signal (CDCl_3_: δ (^1^H) = 7.25 ppm, δ (^13^C) = 77.0 ppm; CD_3_OD: δ (^1^H) = 3.34 ppm, δ (^13^C) = 49.86 ppm) or to the signal of a trace acetone for solutions in D_2_O (δ (^1^H) = 2.22 ppm, δ (^13^C) CH_3_ = 30.9 ppm). Chemical shifts (δ) are reported in ppm and the H-H coupling constants (*J*) are reported in Hz. An Agilent 1100 LC/MSD running with an electrospray source was used in mass spectrometry measurements. The intermediate α,ω-diazido-hexa(ethylene glycol) **1** was synthesized as previously reported in [20].

#### 2.1.1. Grafting Procedure for the Preparation of **HA(270)-FA-Pg** Copolymers

**Figure 3:**
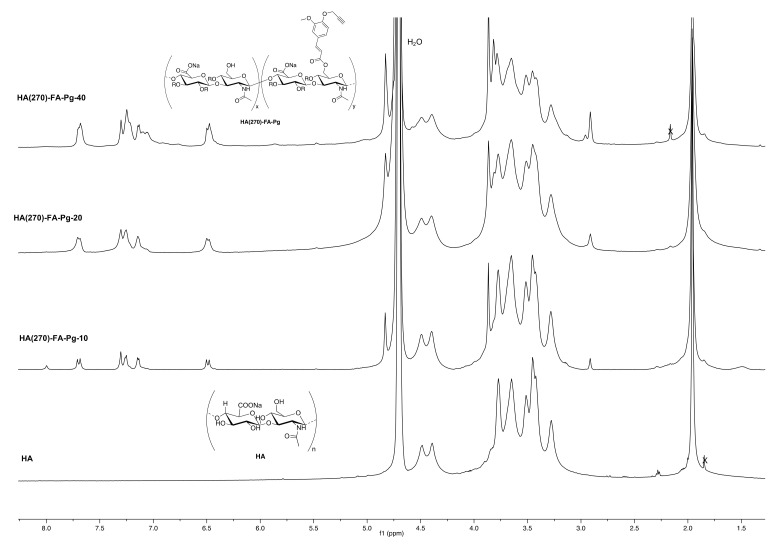
^1^H NMR spectra obtained with the three newly synthesized **HA(270)-FA**-**Pg** graft copolymers (D_2_O, 600 MHz) compared with that obtained with a starting **HA** sample.

A mixture of medium molar mass **HA** (sodium hyaluronate from Biophil Italia SpA, Mw = 270 KDa, 1.0 g, 2.49 mmol in monomeric units) in formamide (10 mL) was heated at 50 °C into a two-necked round-bottomed 50 mL flask until the complete dissolution was obtained. To the resulting solution cooled to room temperature, triethylamine (TEA, 0.37 mL, 2.66 mmol) and **2** [16] (see the amounts in Table 1) were added in rapid sequence. The reaction mixture was homogenized by mechanical stirring and stirred overnight at room temperature. A 5% NaCl solution (5.0 mL) was then added, and the resulting mixture was stirred at room temperature for an additional 15 min. The graft copolymer was isolated by treatment of the mixture with acetone (40 mL) and purified by washing four times with the same solvent. The white solid was dried under reduced pressure to afford the expected **HA(270)-FA-Pg** graft copolymer. ^1^H NMR (600 MHz, D_2_O) (Figure 3).

#### 2.1.2. Click-Chemistry Cross-Linking of **HA-FA-Pg** Graft Copolymers

Under an inert atmosphere, a 10 mL flask was charged with *tert*-butanol (2.0 mL), water (2.0 mL), and a solution of CuSO_4_ pentahydrate (12.5 mg, 0.050 mmol) in 0.50 mL of water. A 1M solution of sodium ascorbate in water (0.50 mL) was then added and, subsequently, 1.0 mL of the resulting mixture was used as the catalyst. A mixture of **1** (see the amounts below) and the appropriate **HA-FA-Pg** graft copolymer (250 mg) in *tert*-butanol (25 mL) and water (25 mL) was treated with the catalyst solution (1.0 mL); the reaction mixture was stirred overnight at room temperature and then concentrated under reduced pressure. Purification of the gel residue by washing with acetone resulted in the corresponding **HA-FA-HEG-CL** material, which was dried under reduced pressure. 

#### 2.1.3. **HA(270)-FA-HEG-CL-10** Material

**Figure 4:**
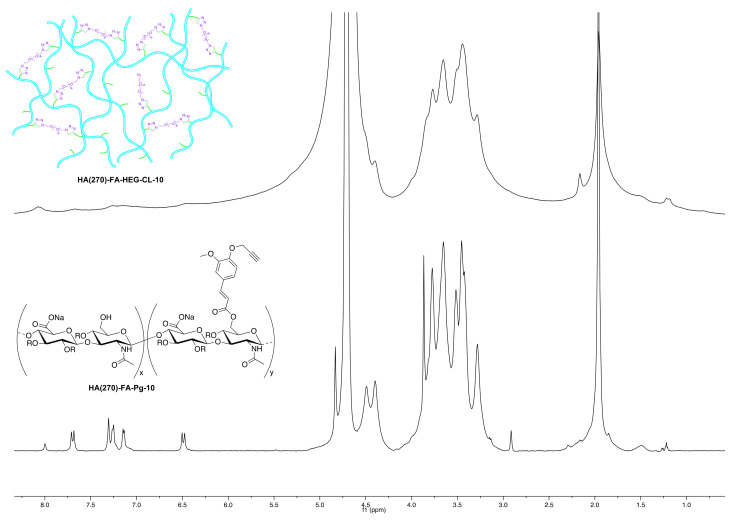
Comparison of the ^1^H NMR spectrum obtained with the cross-linked **HA(270)-FA**-**HEG-CL-10** material (D_2_O, 600 MHz) to that obtained with its corresponding synthetic precursor, the graft copolymer **HA(270)**-**FA**-**Pg**-**10**.

**Figure 5:**
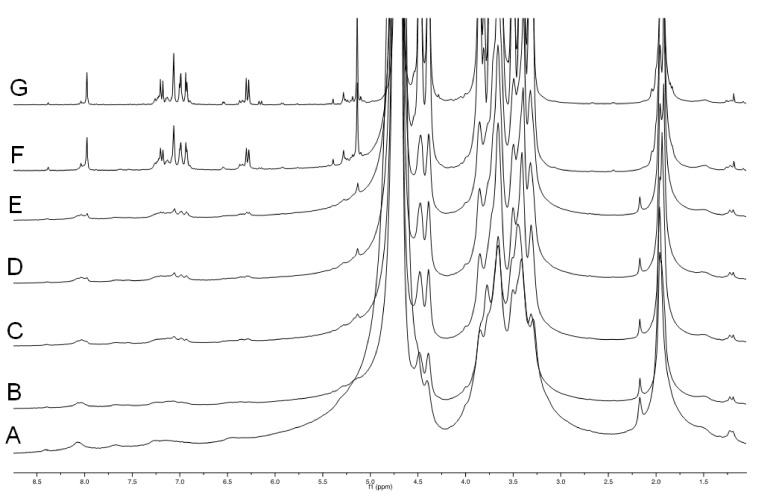
The comparison of ^1^H NMR spectra obtained when a gel dispersion of **HA(270)-FA-HEG-CL**-**10** material in deuterated water was treated with NaOD at room temperature in an NMR tube, and ^1^H NMR spectra were registered at regular time intervals. A: before the sodium hydroxide addition; B: immediately after the sodium hydroxide addition; C: after 10 min at room temperature; D: after 20 min at room temperature; E: after 30 min at room temperature; F: after 90 min at room temperature; G: after 24 h at room temperature.

This cross-linked **HA** derivative was prepared by the above general procedure from **HA(270)-FA-Pg-10** (250 mg) and divalent hexa(ethylene glycol) derivative **1** (10 mg, 0.030 mmol) to obtain **HA(270)-FA-HEG-CL-10** material (246 mg) as a white solid. ^1^H NMR (600 MHz, D_2_O) (Figure 4 and Figure 5A).

#### 2.1.4. **HA(270)-FA-HEG-CL-20** Material

This cross-linked **HA** derivative was prepared using the above general procedure from **HA(270)-FA-Pg-20** (250 mg) and the divalent hexa(ethylene glycol) derivative **1** (23 mg, 0.069 mmol) to obtain **HA(270)-FA-HEG-CL-20** material (256 mg) as an off-white solid. ^1^H NMR (600 MHz, D_2_O) (Figure S1).

#### 2.1.5. **HA(270)-FA-HEG-CL-40** Material

This cross-linked **HA** derivative was prepared using the above general procedure from **HA(270)-FA-Pg-40** (250 mg) and the divalent hexa(ethylene glycol) derivative **1** (41 mg, 0.123 mmol) to obtain **HA(270)-FA-HEG-CL-40** material (280 mg) as a pale yellow solid. ^1^H NMR (600 MHz, D_2_O) (Figure S1).

#### 2.1.6. **HA(8.7)-FA-HEG-CL-20** Material

This cross-linked **HA** derivative was prepared using the above general procedure from **HA-FA-Pg-3F** (250 mg) and the divalent hexa(ethylene glycol) derivative **1** (20 mg, 0.060 mmol) to obtain **HA(8.7)-FA-HEG-CL-20** material (268 mg) as a brown solid. ^1^H NMR (600 MHz, D_2_O) (Figure S1).

#### 2.1.7. Diethyl 3,3′-(((((3,6,9,12,15-Pentaoxaheptadecane-1,17-Diyl)Bis(1*H*-1,2,3-Triazole-1,4-Diyl))Bis(Methylene))Bis(Oxy))Bis(3-Methoxy-4,1-Phenylene))(2*E*,2′*E*)-Diacrylate (**6**)

To a solution of divalent hexa(ethylene glycol) derivative **1** (0.58 g, 1.75 mmol) in dry THF (25 mL) compound **5** (0.92 g, 3.54 mmol), DIPEA (1.52 mL, 8.75 mmol), and CuBr (0.13 g, 0.87 mmol) were added. The reaction mixture was stirred overnight at room temperature under a nitrogen atmosphere and then concentrated under reduced pressure. The resulting residue was partitioned between CH_2_Cl_2_ and a saturated NH_4_Cl solution. The organic layer was dried over sodium sulfate and evaporated under reduced pressure. Purification of the residue by flash chromatography with ethyl acetate resulted in **6** (1.2 g, yield 80%) as an orange oil. ^1^H NMR (300 MHz, CD_3_OD): 1.29 (t, *J* = 7.1, 6H), 3.48 (s, 16H), 3.79 (s, 6H), 3.80–3.84 (m, 4H), 4.19 (q, *J* = 7.1 Hz, 4H), 4.49–4.55 (m, 4H), 5.16 (s, 4H), 6.37 (d, *J* = 16.0, 2H), 7.03–7.11 (m, 4H), 7.17 (d, *J* = 1.6, 2H), 7.57 (d, *J* = 15.9, 2H), 8.08 (s, 2H). MS (ESI): *m/z* 875.4 (M + Na^+^).

#### 2.1.8. Ethyl (*E*)-3-(4-((1-(17-Azido-3,6,9,12,15-Pentaoxaheptadecyl)-1*H*-1,2,3-Triazol-4-Yl)Methoxy)-3-Methoxyphenyl)Acrylate (**7**)

To a solution of divalent hexa(ethylene glycol) derivative **1** (0.64 g, 1.92 mmol) in dry THF (20 mL) compound **5** (0.50 g, 1.92 mmol), DIPEA (1.7 mL, 9.60 mmol), and CuBr (0.14 g, 0.96 mmol) were added. The resulting mixture was stirred overnight at room temperature under a nitrogen atmosphere and then concentrated under reduced pressure. The resulting residue was partitioned between CH_2_Cl_2_ and a saturated NH_4_Cl solution. The organic layer was dried over sodium sulfate and evaporated under reduced pressure. Purification of the residue by flash chromatography with ethyl acetate resulted in **7** (0.13 g, yield 11%) as a yellow oil. ^1^H NMR (300 MHz, CD_3_OD): 1.34 (t, *J* = 7.1, 3H), 3.31–3.35 (m, 2H), 3.53–3.60 (m, 16H), 3.60–3.64 (m, 2H), 3.84 (s, 3H), 3.85–3.89 (m, 2H), 4.22 (q, *J* = 7.1, 2H), 4.56–4.60 (m, 2H), 5.22 (s, 2H), 6.41 (d, *J* = 15.9, 1H), 7.08–7.16 (m, 2H), 7.22 (d, *J* = 1.7, 1H), 7.61 (d, *J* = 16.0, 1H), 8.13 (s, 1H). MS (ESI): *m/z* 615.3 (M + Na^+^).

#### 2.1.9. (2*E*,2′*E*)-3,3′-(((((3,6,9,12,15-Pentaoxaheptadecane-1,17-Diyl)Bis(1*H*-1,2,3-Triazole-1,4-Diyl))Bis(Methylene))Bis(Oxy))Bis(3-Methoxy-4,1-Phenylene))Diacrylic Acid (**3**)

**Figure 6:**
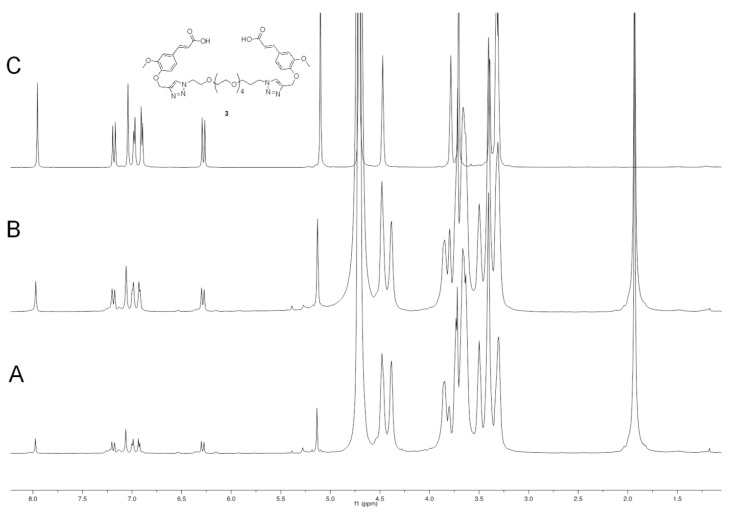
The comparison of ^1^H NMR spectra obtained when a gel dispersion of **HA(270)-FA-HEG-CL**-**10** material in deuterated water was treated with sodium hydroxide at room temperature in an NMR tube for 24 h. (**A**): before the addition of compound **3**; (**B**): immediately after addition of compound **3**; (**C**): ^1^H NMR spectrum of a solution of reference compound **3** in deuterated water containing NaOD.

To a solution of compound **6** (0.89 g, 1.04 mmol) in ethanol (16 mL), a 2 N solution of NaOH (10 mL) was added, and the resulting reaction mixture was heated under reflux for 2 h. Then, the reaction mixture was cooled to 0 °C and 3 N HCl was added dropwise until pH 2, and the crude was extracted with CH_2_Cl_2_. The organic layer was dried over sodium sulfate and evaporated under reduced pressure to give us compound **3** (0.75 g, yield 90.5%) as an off-white solid. ^1^H NMR (600 MHz, CDCl_3_): 3.57 (m, 16H), 3.83 (t, *J* = 5.1, 4H), 3.85 (s, 6H), 4.51 (t, *J* = 4.9, 4H), 5.30 (s, 4H), 6.28 (d, *J* = 15.9, 2H), 7.04 (m, 6H), 7.64 (d, *J* = 15.8, 2H), 7.84 (s, 2H). MS (ESI): *m/z* 819.3 (M + Na^+^). ^1^H NMR (600 MHz, D_2_O-NaOD): Figure 6.

#### 2.1.10. (*E*)-3-(4-((1-(17-Azido-3,6,9,12,15-Pentaoxaheptadecyl)-1*H*-1,2,3-Triazol-4-Yl)Methoxy)-3-Methoxyphenyl)Acrylic Acid (**4**)

To a solution of compound **7** (0.13 g, 0.22 mmol) in ethanol (5.0 mL), a 2 N NaOH solution (2.0 mL) was added, and the resulting mixture was heated under reflux for 2 h. Then, the reaction mixture was cooled to 0 °C and 3 N HCl was added dropwise until pH 2, and the crude was extracted with CH_2_Cl_2_. The organic layer was dried over sodium sulfate and evaporated under reduced pressure to give us compound **4** (0.12 g, yield 97%) as an off-white solid. ^1^H NMR (600 MHz, CDCl_3_): 3.36 (t, *J* = 5.1, 2H), 3.51–3.69 (m, 20H), 3.86 (t, *J* = 5.1, 2H), 3.88 (s, 3H), 4.53 (t, *J* = 5.0, 2H), 5.33 (s, 2H), 6.30 (d, *J* = 15.9, 1H), 7.05–7.09 (m, 3H), 7.67 (d, *J* = 15.9, 1H), 7.86 (s, 1H). MS (ESI): *m/z* 587.2 (M + Na^+^).

### 2.2. SEC-MALS

The characterization of the molecular weight distribution (MWD) was performed by a multi-angle laser light scattering (MALS) detector on-line to a size exclusion chromatography (SEC or GPC) system. The SEC-MALS multi-detector system consisted of an Alliance 2695 chromatograph from Waters (USA) with two on-line detectors: a MALS Dawn DSP-F photometer from Wyatt (USA) and a 410 differential refractometer (DRI) from Waters as a concentration detector. 

SEC experimental conditions were the following: two Shodex OHpak SB (806 HQ–805 HQ, 13 μm of particle size) columns from Showa Denko (J), 35 °C of temperature, 0.8 mL/min of flow rate, and about 2 mg/mL of a sample concentration. Two solvents (0.2 M NaCl + 0.1 M phosphate buffer pH 7.4 and 0.1 M carbonate buffer pH 10) were used as a mobile phase to try to overcome the solubility decrease produced by the grafting increase.

The MALS calibration constant was calculated using toluene as a standard by assuming a Rayleigh Factor of 1.406·10^−5^ cm^−1^. MALS angular normalization was performed by measuring the scattering intensity of a BSA globular protein (M = 66.4 kg/mol, R_g_ = 2.9 nm) assumed to act as an isotropic scatterer. It is known that the on-line MALS detector measures—for each polymeric fraction eluted from the SEC columns—the molecular weight (M), and when the angular dependence of the scattered light is experimentally measurable, the molecular size is also generally known as the radius of gyration (Rg). The SEC-MALS system was described in detail elsewhere [22,23]. The differential refractive index increment of the polymer with respect to the solvent (dn/dc = 0.140 mL/g) was calculated from the area of the DRI concentration detector after accurate calibration.

### 2.3. Swelling Performance

#### 2.3.1. Swelling Kinetics

5 mg of each hydrogel (dry state) were positioned on a cell culture strainer and immersed in bidistilled water at 37 °C. The weight of each sample was monitored until it reached the swelling equilibrium, and the water uptake (WU) was calculated using Formula (1):

WU = [(Ws − Wd/Wd)] · 100(1)

The water content of the hydrogel was calculated using the Formula (2):

WC = [(Ws − Wd)/Ws] · 100(2)

where the Ws and Wd are the weight of swollen and dried hydrogel, respectively.

#### 2.3.2. Total Water

Swollen samples (15–20 mg) were put in a platinum crucible and heated from 30 °C to 250 °C with a rate of 10 °C/min under nitrogen purge gas using a Q600 analyzer (TA Instruments-Waters, New Castle, DE, USA). The percent weight loss in the temperature range 30–120 °C permits quantifying the total water (WH).

#### 2.3.3. Free and Bound Water

Swollen samples (5 mg) were sealed in anodized aluminium pans, cooled to −40 °C, and then heated up (2 °C/min) to 40 °C using a DSC-TA Q 2000 to obtain the percentages of freezable water (free and bound water) following the procedure reported by Li et al. [24]. Briefly, the total weight of water (WH), obtained by TGA, is the sum of freezing (WfH) and non-freezing (WnfH) water weights (3):

WH = WfH + WnfH(3)

DSC thermograms of frozen hydrogels and of bidistilled water (from which the latent heat ΔH was obtained) permit quantifying latent heat ΔH of the freezing water (ΔHm). The freezing water (WfH) was derived using Equation (4):

WfH/Ws = ΔHm/ΔH (4)

where Ws is the weight of the swollen hydrogel.

By the difference non-freezing (WnfH), water weight can be obtained (Equation (3)).

### 2.4. Rheological Analysis

All rheological analyses were carried out on swollen samples at 37 °C using a Discovery HR-2 Rheometer (TA Instruments) equipped with a Peltier steel plate environmental system. Preliminary strain sweep tests were run to identify the Linear Viscoelasticity Region (LVR) recording elastic modulus of the materials at fixed oscillation frequencies (i.e., 0.1 Hz, 1 Hz, and 10 Hz) while varying the strain % from 0.1 to 10% [25].

Elastic (G′) and viscous (G″) moduli of samples (shear strain: 0.25%) were measured as a function of oscillation frequency in the range of 0.1–10 Hz using a plate–plate geometry (Ø = 4 cm).

Viscosity measurements were performed by shear rate experiments (shear ramp from 0.01 to 250 s^−1^) using a 1° cone-plate stainless steel geometry (40 mm, truncation 28.0 μm).

### 2.5. Thermal Behavior

Thermogravimetric Analysis was conducted using a Q600 thermogravimetric analyzer (TA Instruments-Waters, New Castle, DE, USA). Samples (15–20 mg) at the dry state were inserted into a platinum crucible and heated from room temperature to 600 °C (heating ramp 10 °C/min) under nitrogen gas. Samples were set up in three replicates.

### 2.6. Cell Culture and Cytotoxicity Test

In order to evaluate the in vitro cytotoxicity of **HA-FA-HEG-CL** materials, the direct contact tests, proposed by ISO 10995-5, Biological evaluation of medical devices–Part 5: Tests for cytotoxicity: in vitro methods was used [26]. This test is suitable for samples with various shapes, sizes, or physical status (i.e., liquids or solids). The evaluation of in vitro acute toxicity does not depend on the final use for which the product is intended, and the document ISO 10995-5:2009 recommends many cell lines from the American Type Collection. Among them, to test **HA-FA-HEG-CL** cytotoxicity, NIH3T3 mouse fibroblasts were chosen (American Type Culture Collection (USA). Fibroblasts NIH3T3 were propagated in DMEM supplemented with 10% fetal calf serum, 1% L-glutamine-penicillin-streptomycin solution, and 1% MEM non-essential amino acid solution, and then incubated at 37 °C in a humidified atmosphere containing 5% CO_2_. Once at confluence, the cells were washed with 0.1 M PBS, separated with trypsin-EDTA solution, and centrifuged at 1000 r.p.m. for 5 min. The pellet was re-suspended in complete medium (dilution 1:15).

Cells (1.5 × 10^4^) suspended in 1 mL of complete medium were seeded in each well of a 24 well round multidish and incubated at 37 °C in an atmosphere of 5% CO_2_. After 24 h of culture, the culture medium was discharged and the swelled hydrogel (3 mg) was added to each well. The samples were set up in three replicates. Low density polyethylene (LDPE) (U.S. Pharmacopeia (Rockville, MD, USA) was used as the negative control and organo-tin stabilized polyurethane as the positive control (Gradko International Limited, Winchester, UK).

Cell viability was evaluated by Neutral Red uptake after 24, 48, and 72 h of incubation with 3T3 following the procedure previously reported [27].

The analysis of cell morphology after 24 h of incubation was performed via the optical microscopy observation of cells in contact with the test samples by using an inverted, phase-contrast microscope (Olympus IX50) equipped with a video camera (Optika). Reactivity of the test sample was indicated by malformation, degeneration, and the lysis of cells.

## 3. Results and Discussion

### 3.1. Synthesis of **HA(270)-FA-Pg** and **HA-FA-HEG-CL** Derivatives

**Scheme 1:**
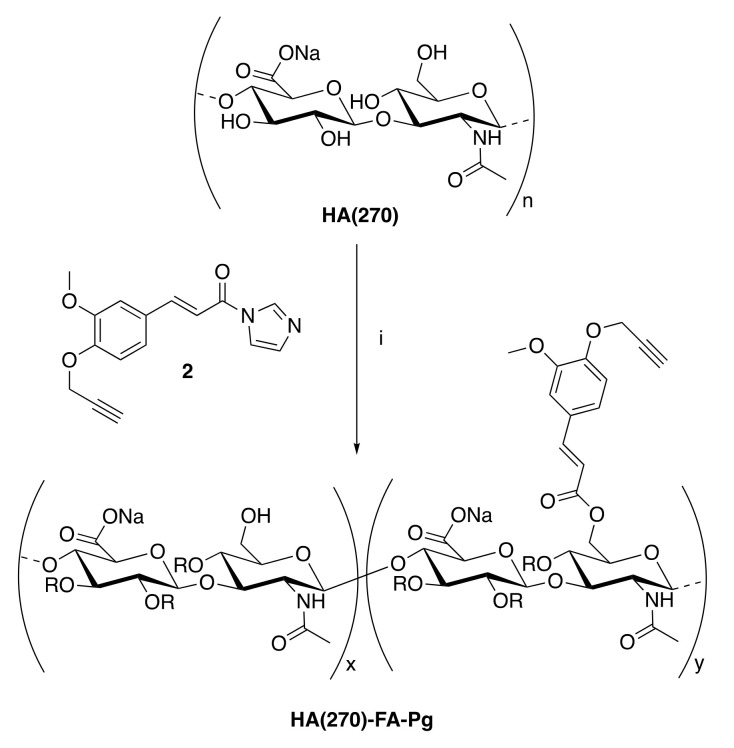
The grafting reaction of medium molar mass **HA**(270) with imidazolide derivative **2** for the preparation of the **HA(270)-FA-Pg** graft copolymers. **Reagents**: (i) HCONH_2_, TEA. **Substituents**: R = H or C_13_H_11_O_3._.

**HA(270)-FA-Pg** graft copolymers were synthesized by reaction of medium molar mass (i.e., Mw = 270 Kda) **HA** with imidazolide derivative **2** [16], as depicted in Scheme 1.

The grafting procedure was performed in the presence of triethylamine (TEA) as the base in formamide as the solvent to obtain the corresponding graft copolymers **HA(270)-FA-Pg**. The stoichiometric ratio between **2** and **HA** (i.e., **2**/**HA** ratio, Table 1) varied from 12.5 to 50%, and the isolation of the copolymers from the reaction mixture was carried out via precipitation with acetone to obtain samples as white solids.

**Table 1:** Reaction parameters in the grafting of **HA(270)** to **HA(270)-FA-Pg** graft copolymers with imidazolide derivative **2**.

Copolymer	HA (g)	HA (mmol)	2/HA Ratio (%)	Grafting Degree ^a^ (%)	Convers. ^b^ (%)
**HA(270)-FA-Pg-10**	1.0	2.49	12.5	10	80
**HA(270)-FA-Pg-20**	1.0	2.49	25	20	80
**HA(270)-FA-Pg-40**	1.0	2.49	50	40	80

^a^ The determination of the grafting degree was made by ^1^H NMR spectroscopy after hydrolysis with NaOD in D_2_O as described in ref. [14]. ^b^ The conversion into ferulate was calculated from the substitution degree and stoichiometric ratio **2**/**HA**.

The data shown in Table 1 suggested that the GD value could be adjusted in the range of 10–40% by employing the correct **2**/**HA** stoichiometric ratios with high conversion values (i.e., 80%).

**Scheme 2:**
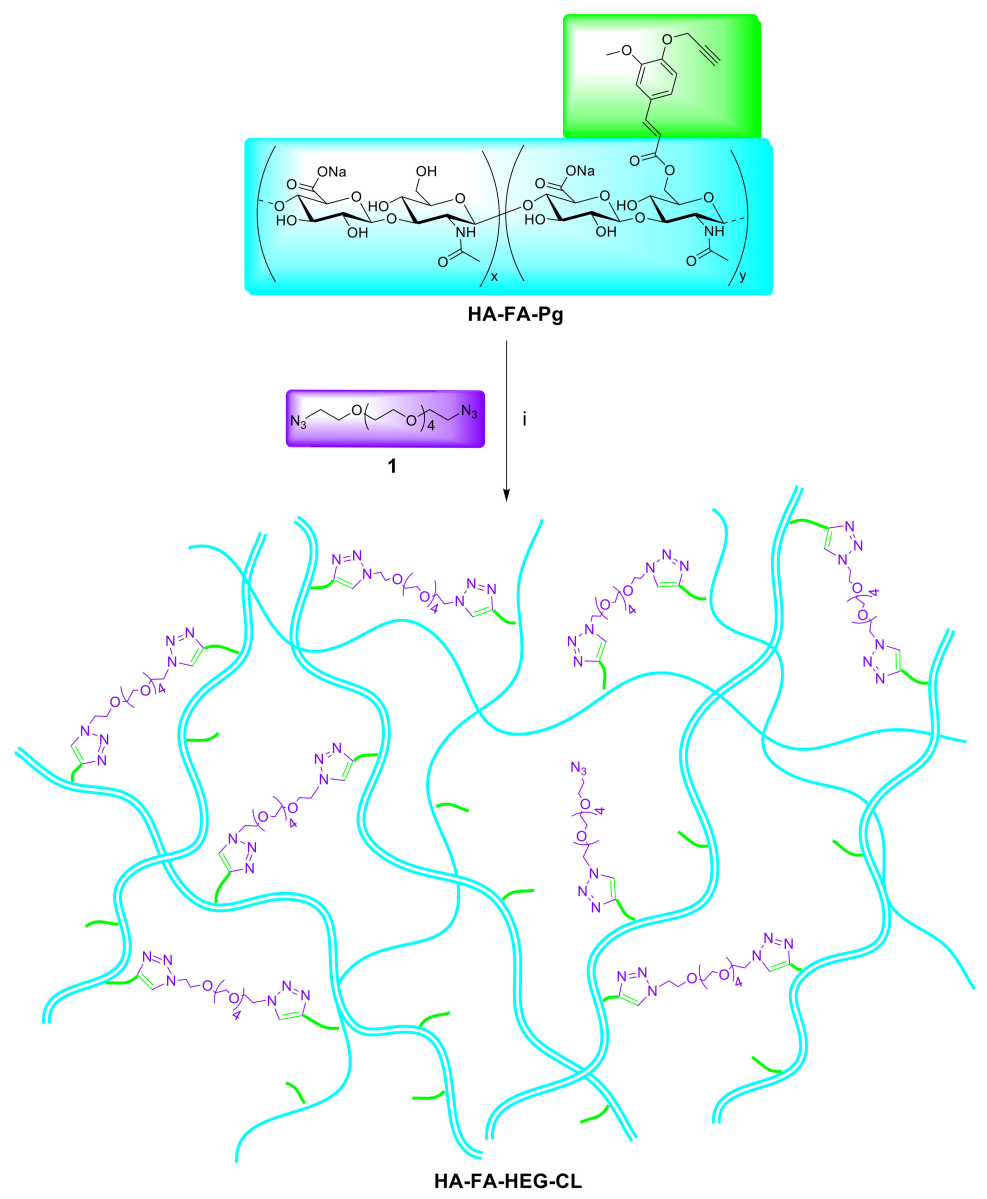
The click chemistry dimerization of **HA-FA-Pg** with divalent azide derivative **1**, leading to **HA-FA-HEG-CL** materials. **Reagents**: (i) CuSO_4_, sodium ascorbate, tert-BuOH, H_2_O.

**HA(270)**-**FA**-**Pg** graft copolymers were then submitted to the CuAAC dimerization reaction with divalent azide derivative **1** (Scheme 2) to obtain cross-linked **HA(270)** derivatives (i.e., **HA(270)-FA-HEG-CL**).

The dimerization reaction was performed by employing the three **HA(270)-FA-Pg** samples showing grafting degree values of 10, 20, and 40% in the aim of obtaining **HA(270)-FA-HEG-CL** derivatives bearing different densities of cross-linking groups affecting the mobility of the **HA(270)** backbone in **HA(270)-FA**-**HEG-CL**-**10**, **HA(270)-FA**-**HEG-CL**-**20**, and **HA(270)-FA**-**HEG-CL**-**40** materials. Thus, the amounts of divalent azide derivative **1** were calculated in order to induce the exhaustive dimerization of the propargylated ferulate residues grafting the **HA** backbones of **HA**-**FA**-**Pg** graft copolymers. As described previously, the catalytic species copper(I) was generated in situ from CuSO_4_ with sodium ascorbate to perform the CuAAC dimerization reaction under very mild conditions.

In order to explore the role of the backbone length, the dimerization reaction was also applied to the previously published **HA-FA-Pg** graft copolymer showing a low molar mass value (i.e., Mw = 8.7 KDa) and a grafting degree of 20% (i.e., **HA-FA-Pg-3F**) to obtain **HA(8.7)-FA-HEG-CL-20** material.

**Scheme 3:**
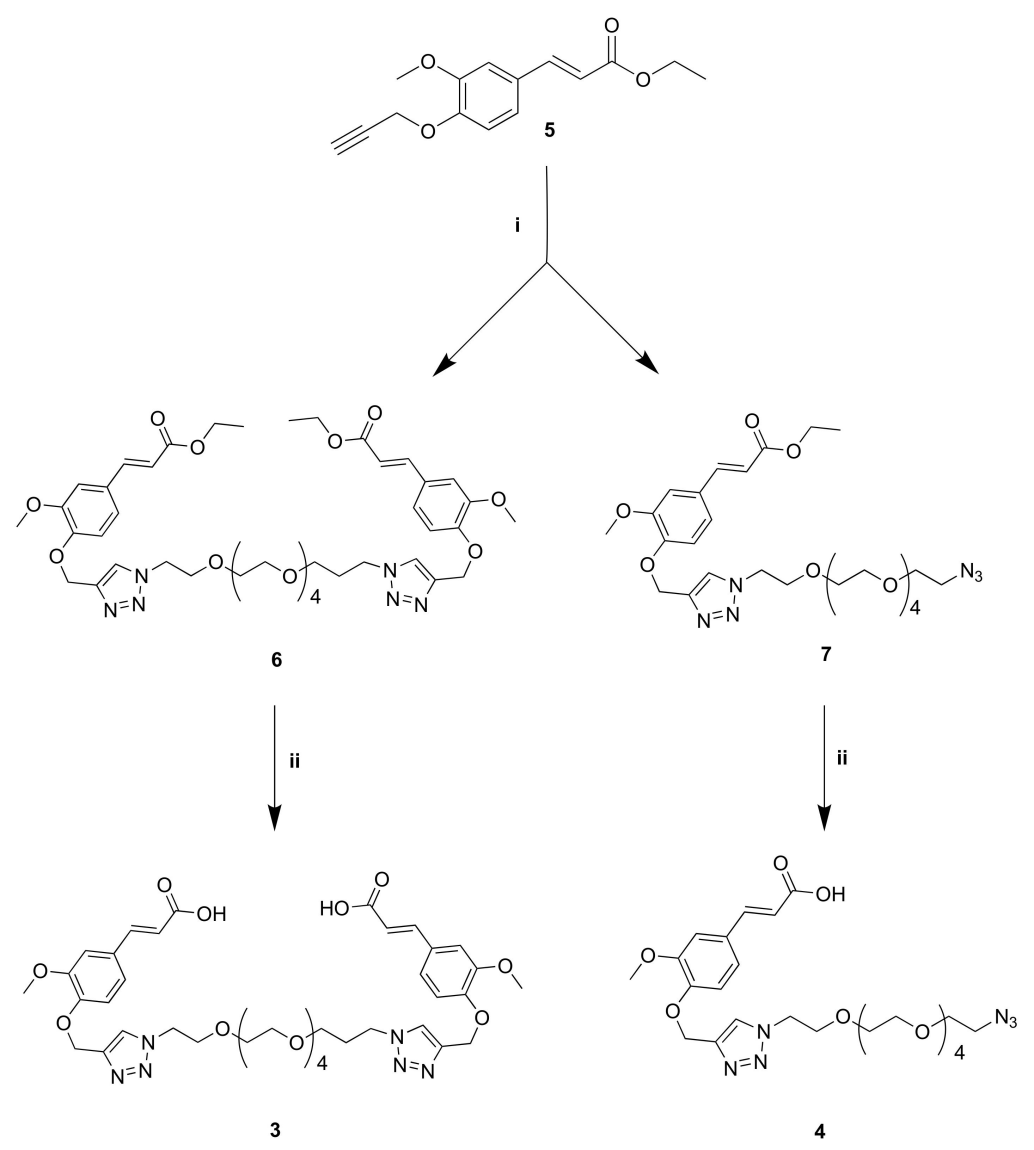
The synthesis of standard compounds **3** and **4**. **Reagents:** (i) compound **1**, CuBr, DIPEA, THF; (ii) 2N NaOH, H_2_O, C_2_H_5_OH.

Finally, compounds **3** and **4** were synthesized as described in Scheme 3 to be used as standard compounds in hydrolysis studies.

Compounds **3** and **4** and their respective ethyl ester precursors **6** and **7** were synthesized from the intermediate ferulate **5** bearing a propargyl group [28] and an hexa(ethylene glycol) derivative terminated with azide groups at both ends (compound **1**, azido-**HEG-**azido) [20]. In particular, a copper(I)-catalyzed azide alkyne 1,3-dipolar cycloaddition (CuAAC) reaction was performed using copper(I) bromide as a catalyst in the presence of *N*,*N*-diisopropylethylamine (DIPEA) as the base in THF. The stoichiometric ratio of the reaction between propargylated ferulate **5** and compound **1** was optimized in order to obtain the divalent derivative **6** and the monovalent derivative **7** with different yields. Nevertheless, while the former was achieved with good conversion value (i.e., 80%), the latter was obtained with low yield (i.e., 11%) due to the fact that the reaction mainly evolved towards the formation of the divalent derivative **6**. Then, it was necessary to hydrolyze the ester group of compounds **6** and **7** with 2 N NaOH solution in ethanol to obtain the respective compounds **3** and **4** with very good yields to be used as standards in hydrolysis studies.

### 3.2. Structure of **HA-FA-Pg** and **HA-FA-HEG-CL** Derivatives

The structure of newly prepared **HA-FA-Pg** and **HA-FA-HEG-CL** derivatives was researched by ^1^H NMR spectroscopic studies using D_2_O as the solvent. The comparative analysis of the ^1^H NMR spectra obtained with the three **HA(270)**-**FA**-**Pg** graft copolymers (Figure 3) substantiated the efficacious link between **HA** and **1** in the conditions used in the grafting reaction.

As expected, the ^1^H NMR spectra of the graft copolymers showed, in their down-field region, signals attributed to the propargylated **FA** residues. The relative intensity of these signals increased from **HA(270)-FA-Pg**-**10** (grafting degree around 10%) to **HA(270)-FA-Pg**-**20** (grafting degree around 20%) and **HA(270)-FA-Pg**-**40** (grafting degree around 40%), paralleling the increase of the grafting degree. Furthermore, the broadness of the signal pattern appearing in the aromatic region was considered to be a further support of the effective coupling. The comparative analysis of the aromatic peaks at around 7.2 ppm suggested the presence of structural inhomogeneities, which appeared to increase with the increase in grafting degree. This observation was interpreted in terms of different sites of functionalization in addition to the most accessible primary OH groups. Moreover, the presence of a very small singlet at around 8.0 ppm may be related to the presence of formyl groups deriving from the formamide used as the reaction solvent.

In order to evaluate the occurrence of the click chemistry cross-linking of **HA(270)**-**FA**-**Pg** graft copolymers (i.e., **HA(270)-FA-Pg-10**, **HA(270)**-**FA**-**Pg**-**20**, and **HA(270)**-**FA**-**Pg**-**40**) leading **HA(270)**-**FA**-**HEG-CL** derivatives (i.e., **HA(270)**-**FA**-**HEG-CL**-**10**, **HA(270)-FA**-**HEG-CL**-**20**, and **HA(270)-FA**-**HEG-CL**-**40**, respectively), the ^1^H NMR spectrum of **HA(270)-FA**-**HEG-CL**-**10** was analyzed in comparison to that of its corresponding synthetic precursor, the graft copolymer **HA(270)**-**FA**-**Pg**-**10** (Figure 4).

As expected, the disappearance of the singlet signal at around 2.91 ppm assigned to the alkyne hydrogen atom of **HA(270)**-**FA**-**Pg**-**10** graft copolymer and the presence of a new singlet at around 8.1 ppm in the spectrum of **HA(270)-FA**-**HEG-CL-10** material supported the conversion of the alkyne portion into the triazole one, as it usually happens in a CuAAC coupling reaction. Thus, this observation was taken into account as a strong structural evidence concerning **HA(270)-FA**-**HEG-CL-10** material. Moreover, the remarkable line broadening affecting all the peaks in the spectrum of **HA(270)-FA**-**HEG-CL-10** material is strongly suggestive of the reduced mobility in the cross-linked macromolecules of this material.

With the aim of identifying the actual cross-linking species, a gel dispersion of **HA(270)-FA**-**HEG-CL-10** material in (deuterated) water was treated with sodium hydroxide at room temperature, and a rather rapid gel–sol transition was observed. When the same experiment was performed in an NMR tube and ^1^H NMR spectra were registered at regular time intervals, we obtained the results summarized in Figure 5.

The results shown in Figure 5 suggest that the presence of sodium hydroxide is capable of promoting the cleavage of the ester bond linking ferulate residues to the **HA** backbone with the formation of a major ferulate derivative and 2–3 minor products.

In order to identify the major product of the hydrolysis, dimeric compound **3** was added to the hydrolysis mixture, the result of which is shown in Figure 6.

Figure 6 shows that dimeric compound **3** is the major product of hydrolysis, and as expected, this compound is responsible for the cross-linking in **HA(270)-FA-HEG-CL**-**10** when covalently bound to **HA** hydroxy groups. In fact, when compound **3** was added to the hydrolysis mixture in the NMR tube, a significant increase was observed in the intensity of the signals attributed to the major product. Very similar results were obtained with **HA(270)-FA-HEG-CL**-**20** material, but the ^1^H NMR hydrolysis experiments performed with **HA(270)-FA-HEG-CL**-**40** material suggested that the cross-linking of the highly grafted **HA(270)**-**FA**-**Pg**-**40** copolymer was not exhaustive (Figure S2). We assumed that during the cross-linking process, the increasing rigidity of the partially cross-linked material prevents the exhaustive combination of all the clickable groups present in the highly grafted copolymer. Thus, with the progress of the cross-linking process leading to **HA(270)-FA-HEG-CL**-**40** material and the increase in rigidity, divalent azide derivative **1** is capable of performing only the first CuAAC reaction, but not the complete dimerization.

### 3.3. Molecular Characterization of **HA(270)-FA-Pg** Derivatives

The characterization of the macromolecular features of **HA(270)-FA-Pg** graft copolymers was performed in comparison of the starting **HA** by means of a multi-angle laser light scattering (MALS) absolute detector on-line to a size exclusion chromatography (SEC) system. Two solvent systems (i.e., 0.2 M NaCl + 0.1 M phosphate buffer pH 7.4 or 0.1 M carbonate buffer pH 10) were employed as the mobile phases to circumvent the solubility decrease produced by the increase in the grafting degree. Comparable results were obtained for **HA(270)-FA-Pg-10** and **HA(270)-FA-Pg-20** in the two solvent systems, and **HA(270)-FA-Pg-40** was difficult to be analyzed at both pH values. The best results were obtained by using carbonate buffer at pH 10, and SEC-MALS results such as the molecular weight of the peak of the chromatogram (*M*_p_), the weight-average of the molecular weight (*M*_w_), and the polydispersity index *M*_w_/*M*_n_ (where *M*_n_ denotes the numeric-average of the molecular weight) are summarized in Table 2.

The molecular weight of the starting **HA(270)** sample was in the expected range, and the molecular weight distribution was substantially broad (*M*_w_/*M*_n_ about 3.9). The Recovered Mass values of the samples showing low grafting degrees were relatively high and adequate for estimating the molecular weight distribution of those graft copolymers after the derivatization reactions.

Thus, the grafting reaction leading to **HA(270)-FA**-**Pg-10** and **HA(270)-FA**-**Pg-20** derivatives produced only small increases in molar weight of the macromolecules (i.e., the *M*_w_ average increased from 274 kg/mol of the starting **HA(270)** to 330 kg/mol of the **HA(270)-FA**-**Pg-10** derivative showing 10% of grafting or to 387 kg/mol of the **HA(270)-FA**-**Pg-20** derivative showing 20% of grafting).

The derivatization reaction can be assumed to produce two different effects on molecular weight distributions that can work simultaneously in opposite ways. The most obvious effect is the molecular weight increase produced by the grafting, while the less obvious, but however possible effect, is the molecular weight decrease produced by an eventual degradation. The results summarized in Table 2 suggest that the degradation of the starting **HA(270)** biopolymer is also very low for a high level of grafting.

Unfortunately, the molecular weight data (Table 2) are influenced by the insolubility increases when the grafting degree increases. Specifically, the low Recovered Mass value of the **HA(270)-FA-Pg-40** sample (≈21%) means that the molecular weight of this sample is probably underestimated because the higher molecular weight fractions are not soluble.

### 3.4. Swelling Performance

The swelling performance of a polymeric hydrogel is strictly related to its capability to bind water molecules. Water content (WC) and water uptake (WU) were quantified. All hydrogels reached the swelling equilibrium within 24 h. The water uptake decreased from 8600 recorded for **HA(270)-FA-HEG-CL**-**10** to 2600 for **HA(270)-FA-HEG-CL**-**40,** with sample **HA(270)-FA-HEG-CL**-**20** showing an intermediate WU value of 2800. The obtained values highlighted a significant difference between sample **HA(270)-FA-HEG-CL-10** and the two samples with a higher grafting degree, whereas no significant difference was found in terms of WU between **HA(270)-FA-HEG-CL-20** and **HA(270)-FA-HEG-CL-40**. WC showed the same trend. This observation supported the assumption that **HA(270)-FA-HEG-CL-20** and **HA(270)-FA-HEG-CL-40** have a similar crosslinking degree despite the different grafting degree. **HA(8.7)-FA-HEG-CL**-**20** showed the highest values of WU and WC, reaching 9780 and 99%, respectively, thus suggesting a very soft matrix.

The swelling process can be schematized as a three-step process. During the first phase, water molecules strictly bind to the hydrophilic sites of the polymer. These water molecules that show a limited mobility are uniformly distributed along the polymer skeleton (non freezing water: nfw). After the formation of this first hydration layer, other water molecules layered above the first water layer to form the maximum amount of hydrogen bonds. This second layer showing a larger mobility can be frozen (freezing bound water: fbw). Finally, during the third step, all of the network is fulfilled by water (bulk water, i.e., free freezing water: ffw) [29]. Consequently, the quantification of the three species into which water can be subdivided is of outmost importance.

**Figure 7:**
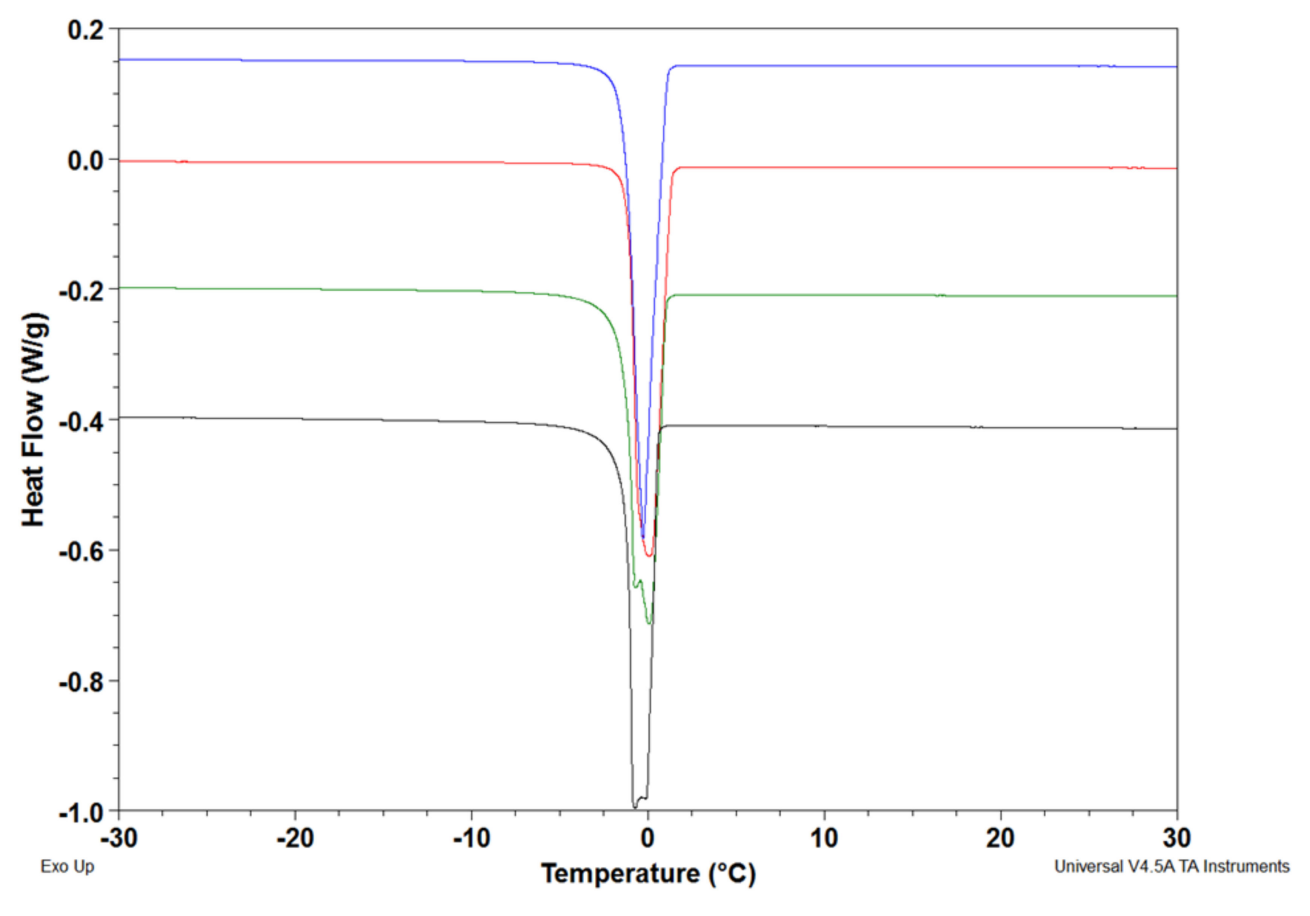
**The** DSC endothermic melting profile of frozen completely swollen hydrogels obtained with **HA(270)-FA-HEG-CL**-**20** (green), **HA(270)-FA-HEG-CL**-**40** (black), **HA(270)-FA-HEG-CL**-**10** (red), and **HA(8.7)-FA-HEG-CL**-**20** (blue).

The presence of a double peak for totally swollen hydrogels (Figure 7) permitted subdividing the freezable water into bound freezing water (Wbf) and free freezing water (Wff), respectively. All the data are reported in Table 3.

The used protocol, as reported in the experimental section, permits quantifying the freezing water and by difference with the total water, quantified by TGA, obtaining the not freezing water (Wnf). As shown by data summarized in Table 3, no significant differences can be observed among the samples in terms of water type distribution. Indeed, the highest percentage is represented by the free freezing water which ranges from 87% found for **HA(270)-FA-HEG-CL**-**20** to 95% found for **HA(8.7)-FA**-**HEG-CL**-**20**. Among the 270 kDa series, accordingly with the WC and WU measurements, **HA(270)-FA-HEG-CL**-**10** shows the highest percentage of Wf and consequently the lowest percentage of not freezable water or the water strictly bound to the matrix polymeric chains (7% against 10–13% found for the hydrogels with a higher grafting degree). Despite the lower grafting degree, **HA(270)-FA-HEG-CL**-**20** showed a lower amount of Wf, and consequently a higher percentage of not freezable water, in comparison with **HA(270)-FA-HEG-CL**-**40.** This phenomenon can be due to a higher crosslinking density in **HA(270)-FA-HEG-CL**-**20** with respect to **HA(270)-FA-HEG-CL**-**40** despite the lower grafting degree. Indeed, as also suggested by ^1^H NMR hydrolysis experiments (Figure S2), a higher grafting degree does not guarantee a higher crosslinking density, since the achievement of an efficient crosslinking degree is possible according to an adequate chain mobility.

### 3.5. Rheological Analysis

**Figure 8:**
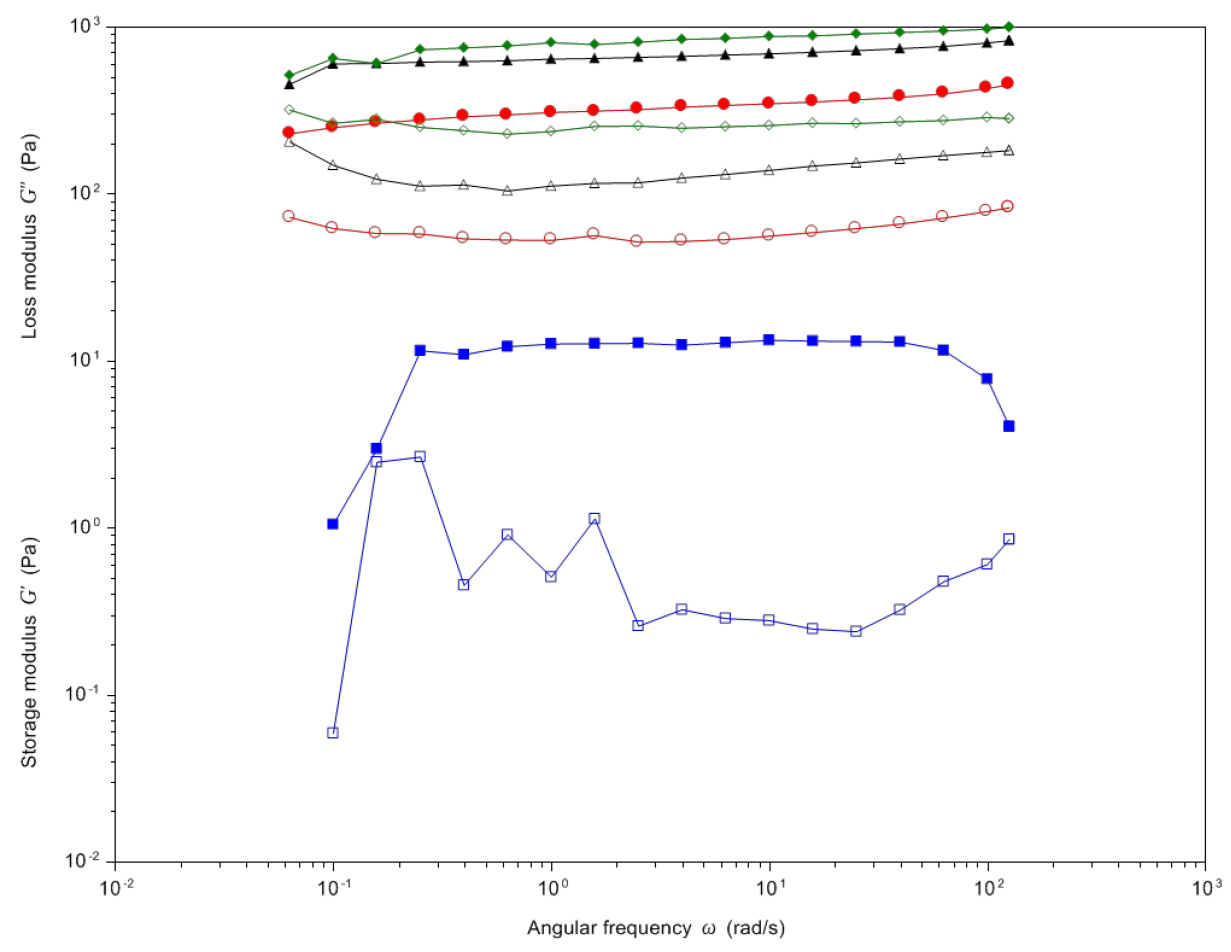
The storage (G′: full symbols) and loss (G″: empty symbols) moduli trend in the angular frequency range of 0.1–100 rad/s of the hydrogels obtained with **HA(270)-FA-HEG-CL**-**20** (green), **HA(270)-FA-HEG-CL**-**40** (black), **HA(270)-FA-HEG-CL**-**10** (red), and **HA(8.7)-FA-HEG-CL**-**20** (blue).

The mechanical properties of **HA(270)-FA-HEG-CL** and **HA(8.7)-FA-HEG-CL**-**20** samples were studied by measuring the storage (G′) and the loss (G″) moduli as a function of frequency. The samples were allowed to relax before starting with a shear test. The frequency sweep test evidenced a “gel-like” behavior for all the tested materials since all of them showed that the storage modulus (G′) was greater than the loss modulus (G″). Moreover, **HA(270)-FA-HEG-CL** series can be defined as strong gels since both moduli are independent from frequency. Among the **HA(270)** series, no significant differences were recorded between **HA(270)-FA-HEG-CL**-**20** and **HA(270)-FA-HEG-CL**-**40**, whereas **HA(270)-FA-HEG-CL**-**10** showed significant lower mechanical properties (Figure 8). Nevertheless, the strongest effect is noted to be molecular weight. Indeed, **HA(8.7)** derivative showed about two orders of magnitude lower moduli values. Moreover, a higher dependence of moduli from frequency could be observed, thus highlighting a lower stiffness for the 8.7 kDa sample in comparison with the 270 kDa series. The stiffness of a material is strictly related to its crosslinking degree, and the mechanical analysis can be used to calculate the average mesh size (ξ) or the crosslinking density.

The average mesh size (ξ), defined as the distance between valid crosslinking points [30], was calculated using the elastic modulus value (G′). The higher the mesh size, the lower the crosslinking density. The hydrogels obtained with **HA(270)-FA-HEG-CL**-**20** and **HA(270)-FA-HEG-CL**-**40** samples showed superimposable mesh size values (i.e., 16 ± 1 nm, 18 ± 2 nm, respectively), whereas for **HA(270)-FA-HEG-CL**-**10** a value of 23 ± 1 nm was found. Finally, sample **HA(8.7)-FA-HEG-CL**-**20** showed a significantly different value of mesh size reaching 68 nm, thus confirming the swelling behavior results.

The obtained results appeared to confirm a slightly higher crosslinking density for **HA(270)-FA-HEG-CL**-**20** in comparison to **HA(270)-FA-HEG-CL**-**40,** which represented a heterogeneous material characterized by a non-exhaustive crosslinking, as suggested by ^1^H NMR hydrolysis experiments (see Figure S2).

**Figure 9:**
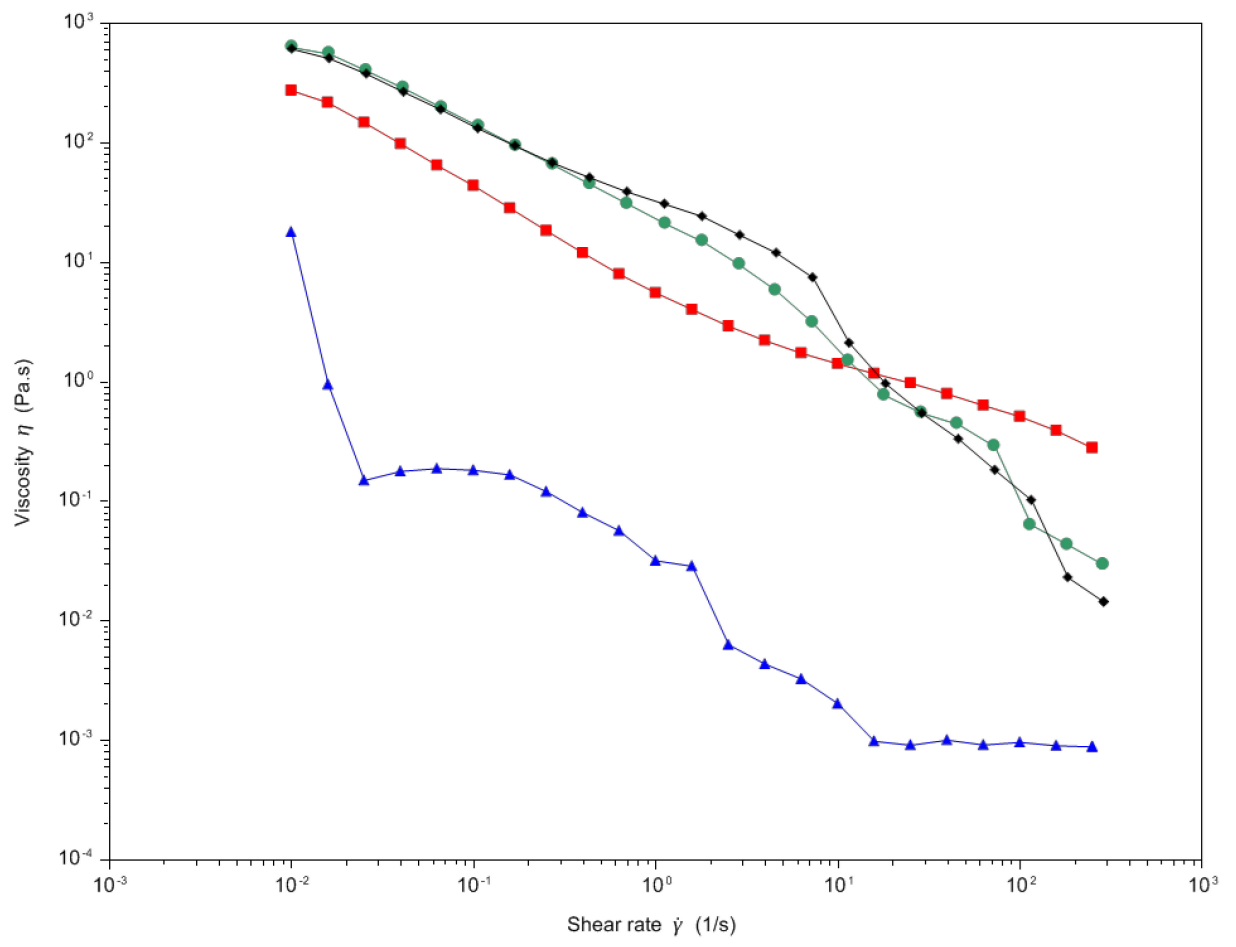
The viscosity curve of the swollen hydrogels obtained with **HA(270)-FA-HEG-CL**-**20** (green), **HA(270)-FA-HEG-CL**-**40** (black), **HA(270)-FA-HEG-CL**-**10** (red), and **HA(8.7)-FA-HEG-CL**-**20** (blue).

In Figure 9, viscosity curves of all the swollen hydrogels are depicted. Similar to swelling performance and viscoelastic properties, the highest effect is molecular weight, in opposition to crosslinking density, as has been already observed for other polymer based hydrogels [31]. Samples **HA(270)-FA-HEG-CL**-**20** and **HA(270)-FA-HEG-CL**-**40** showed a superimposable behavior which was slightly different from **HA(270)-FA-HEG-CL**-**10.** Viscosity curves were consistent with the different stiffness of analyzed materials. Indeed, the higher the crosslinking degree, the lower the resilience of the materials or resistance to shear rate; thus, viscosity can change more abruptly.

The zero shear viscosity was approximated by applying a Cross model that perfectly fit with experimental data (1.6 × 10^6^ Pa·s, R^2^ = 1.000 for **HA(270)-FA-HEG-CL**-**10**; 694 Pa·s, R^2^ = 0.99 for **HA(270)-FA-HEG-CL**-**20**; 410 Pa·s, R^2^ 0.98 for **HA(270)-FA-HEG-CL**-**40**; 1.8 × 10^6^ Pa·s, R^2^ = 0.93 for **HA(8.7)-FA-HEG-CL**-**20**). Viscosity values of the three **HA(270)-FA**-**Pg** graft copolymers are summarized in Table S1. The major mechanical parameters of the hydrogels are summarized in Table 4.

### 3.6. Thermal Behavior

**Figure 10:**
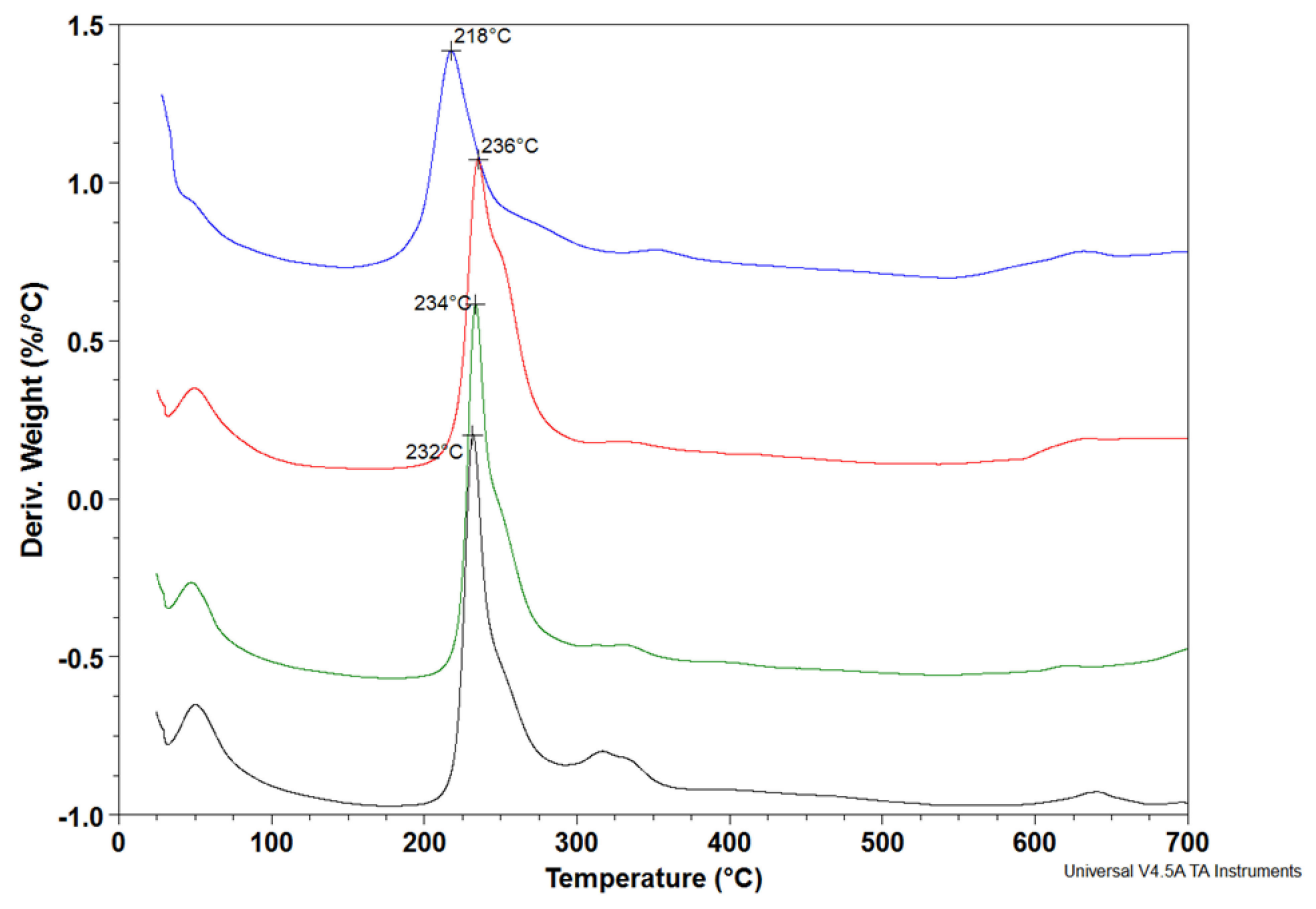
Thermographs obtained plotting the derivative of weight versus temperature (30–600 °C) of the hydrogels obtained with **HA(270)-FA-HEG-CL**-**20** (green), **HA(270)-FA-HEG-CL**-**40** (black), **HA(270)-FA-HEG-CL**-**10** (red), and **HA(8.7)-FA-HEG-CL**-**20** (blue).

Thermographs of all analyzed samples are reported in (Figure S2). Thermographs were obtained by plotting the derivative of weight versus temperature (Figure 10). Three regions of weight loss can be analyzed. The first region that ranges from 30 to 200 °C is related to the evaporation of water bound to the material. The second region spans from 200 °C to 400 °C and can be related to the degradation of free carbon chains. Finally, the third region (400–600 °C) is related to the backbone cleavage (carbonation phase). All weight percent losses are summarized in Table 5. No significant differences were observed among the 270 kDa series. Sample **HA(8.7)-FA-HEG-CL**-**20** shows a similar thermal behavior. Nevertheless, it shows a higher capability to bind water, as pointed out by the high weight per cent loss in the first range of heating. This phenomenon can be explained by considering the very large mesh size of **HA(8.7)-FA-HEG-CL**-**20** in comparison to the hydrogel obtained by the 270 kDa series. The higher the mesh size, the higher the percentage of absorbed water, as also verified by swelling measurements.

**Table 5:** **The** TGA/DTG analysis of samples. Data were reported as percent mean value ± SD (*n* = 3).

Sample	30–200 °C (%)	200–400 °C (%)	400–600 °C (%)
**HA(270)-FA-HEG-CL-10**	13 ± 1	43 ± 3	7 ± 1
**HA(270)-FA-HEG-CL-20**	15 ± 2	43 ± 3	7 ± 1
**HA(270)-FA-HEG-CL-40**	18 ± 2	46 ± 2	7 ± 2
**HA(8.7)-FA-HEG-CL-20**	22 ± 3	43 ± 2	8 ± 2

**HA-FA-HEG-CL** derivatives obtained by crosslinking with CuAAC click chemistry reaction showed a significant decrease of the temperature (about 10 °C) at which the main weight loss occurred in comparison with the native polymers (data not shown). 270 kDa series shows a similar thermal behavior with the main weight loss recorded at about 230 °C despite the crosslinking density. A contrary sample **HA(8.7)-FA-HEG-CL**-**20** degrades at significantly lower temperatures (i.e., 218 °C), according to its lower structuring.

### 3.7. In Vitro Cytotoxicity: Cell Viability and Morphology

**Figure 11:**
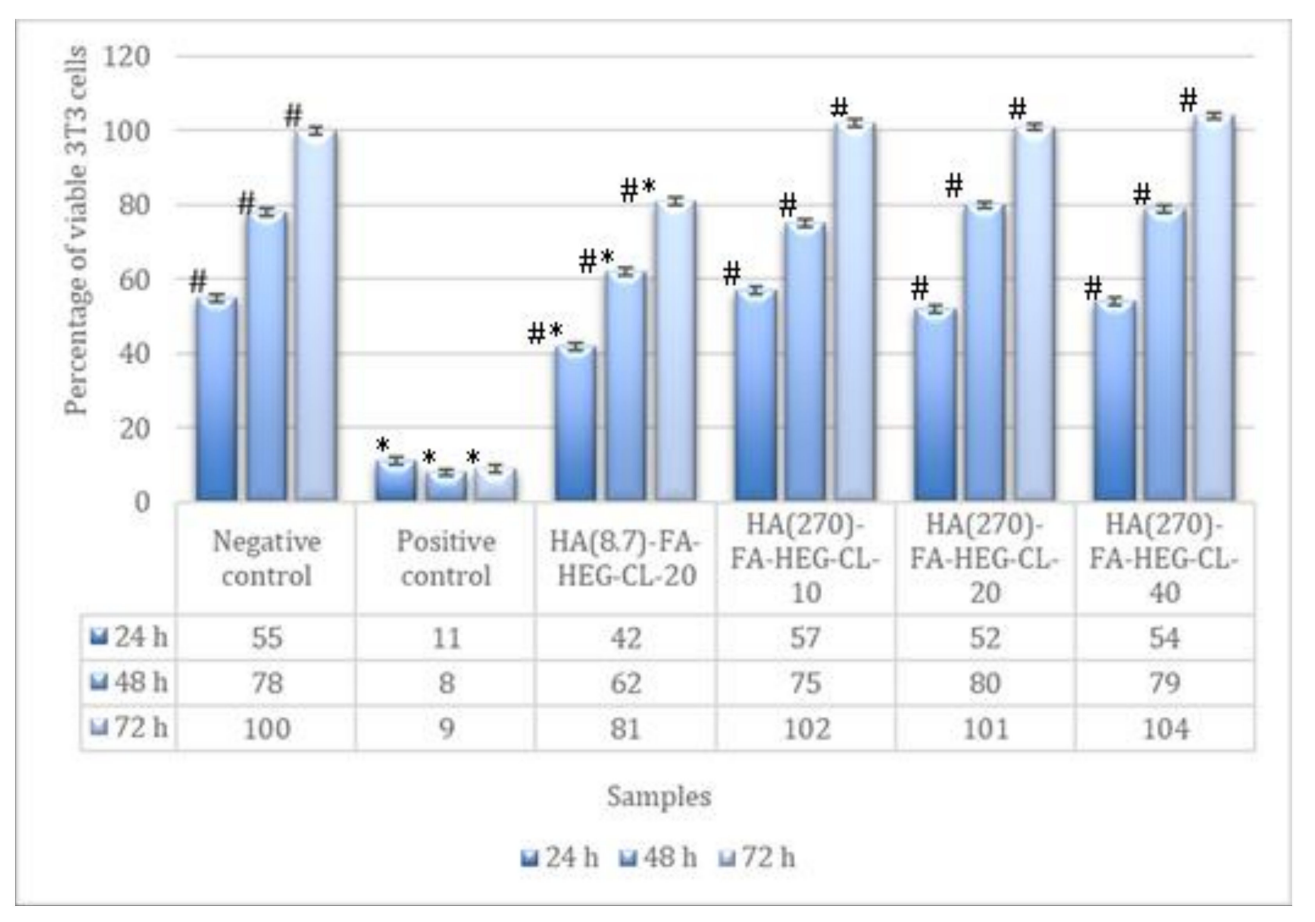
The percentage of viable NIH3T3 fibroblasts evaluated by the neutral red assay after 24, 48, and 72 h of contact with the tested samples (samples concentration = 0.6 mg/cm^2^). Data are means ± SD of one experiment run in triplicate. * Value is statistically different in comparison to negative control LDPE, *p* < 0.05. # Values are statistically different in comparison to positive control PVC-org. Sn, *p* < 0.05.

**Figure 12:**
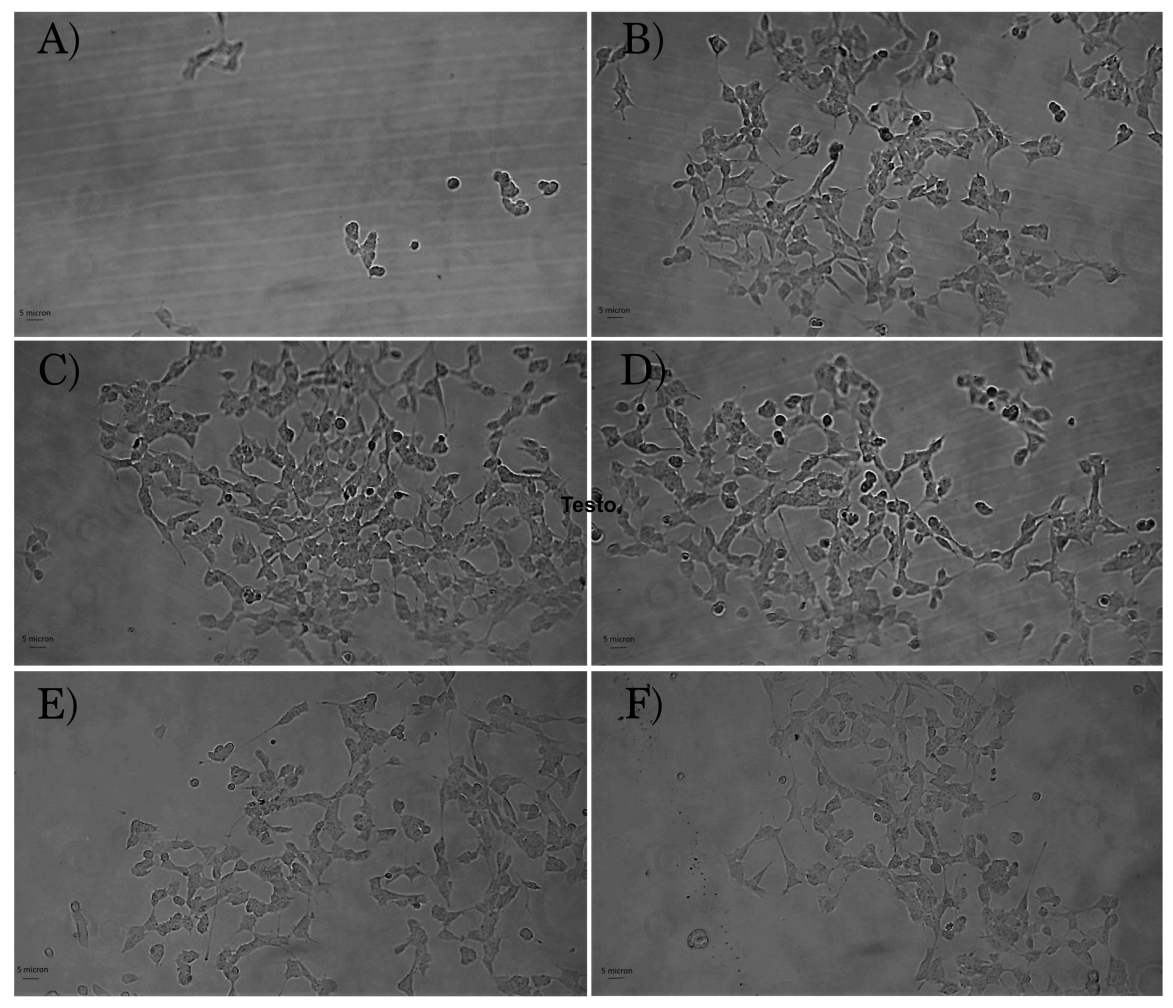
Optical microscope images of NIH3T3 mouse fibroblasts after 24 h of contact with: (**A**) positive control; (**B**) negative control (LDPE); (**C**) **HA(270)-FA-HEG-CL-10**; (**D**) **HA(270)-FA-HEG-CL-20**; (**E**) **HA(270)-FA-HEG-CL-40**; (**F**) **HA(8.7)-FA-HEG-CL**-**20**. Databar 5 µm. Magnification: 20×.

Non-confluent adhered fibroblasts were incubated with a concentration of 0.6 mg/cm^2^ of each test sample. Cells viability and morphology were determined after 24 h of contact, the results of which are reported in Figure 11 and Figure 12.

As shown in Figure 11, **HA(270)-FA-HEG-CL-10**, **HA(270)-FA-HEG-CL-20**, and **HA(270)-FA-HEG-CL-40** demonstrated no toxic effect toward fibroblasts for all three incubation times as the percentage of cells in contact with the test materials was not statistically different (*p* < 0.05) in comparison to the negative control (LDPE), but differed significantly from those of the positive control which had a strong cytotoxic effect. On the contrary, fibroblasts in contact with **HA(8.7)-FA-HEG-CL-20** demonstrated a reduction of cell viability of about 20% in comparison to the negative control for all the incubation times.

Concerning the cytotoxicity, the standard ISO 10993-5 claims that a material can be considered as not cytotoxic if the solid material allows for a cell viability of over 70% after an exposure for 24 h. So, although the **HA(8.7)-FA-HEG-CL-20** sample affected the percentage of cell viability in comparison to the negative control, it cannot be considered cytotoxic, as well as all the other tested samples (i.e., **HA(270)-FA-HEG-CL-10**, **HA(270)-FA-HEG-CL-20**, and **HA(270)-FA-HEG-CL-40**).

As cell shape is closely related to cell function [32], the analysis of cell morphology is an important index of materials cytotoxicity. Optical microscopy images of fibroblasts 3T3 after 24 h of contact with positive control, negative control, and with the tested samples are reported in Figure 12. Cells in contact with the LDPE (Figure 12B) and with all the four tested samples showed the same shape and appeared to be flattened, elongated with a spindle-shaped morphology, confirming the good compatibility of all the materials toward fibroblasts 3T3 (Figure 12c–f).

## 4. Conclusions

In conclusion, a click-chemistry crosslinking (click-crosslinking) procedure was developed to obtain cross-linked **HA** derivatives to be used in the formation of hydrogels. In particular, the clickable propargyl groups of **HA**-**FA**-**Pg** graft copolymers showing low and medium molecular weight values were employed in crosslinking by click-chemistry through a biocompatible hexa(ethylene glycol) spacer as an example of another possible application of our technology platform based on **HA**-**FA**-**Pg** graft copolymers. A short series of medium weight **HA**-**FA**-**Pg** graft copolymers was synthesized, characterized, and used in a CuAAC dimerization reaction under very mild conditions in the presence of very low amounts of copper(I) catalyst. The dimerization reaction was also applied to the previously published **HA-FA-Pg-3F** graft copolymer showing a low molar mass value, and hydrolysis studies confirmed the importance of the dimerization reaction in the formation of cross-linked **HA-FA-HEG-CL** materials. The interaction of the resulting **HA-FA-HEG-CL** materials with water led to the formation of hydrogels showing a wide range of gelation features thanks to the possibility of tuning the crosslinking degree. As a consequence of that, different rheological behaviours have been observed. The tuneable rheological behaviour of **HA-FA-HEG-CL** materials led to their applicability in different biomedical fields. In particular, **HA(270)-FA-HEG-CL-10** seems to be a good candidate for potential application in the treatment of osteoarticular diseases since its shear-thinning ratio perfectly falls in the range of a healthy synovial fluid shear-thinning ratio (70–250). Moreover, the obtained results allow us to also conjecture their suitability as viscosity enhancers for eye drops. Finally, the data of both qualitative and quantitative cytotoxicity resulting from the experiments (performed according to the UNI EN ISO 10993-5 standards) demonstrates that our click-chemistry cross-linking procedure of **HA(270)-FA-Pg** graft copolymers is fully biocompatible since the resulting **HA(270)-FA-HEG-CL-10**, **HA(270)-FA-HEG-CL-20**, and **HA(270)-FA-HEG-CL-40** materials resulted in the avoidance of any in vitro cytotoxic effects.

## Figures and Tables

**Table 2:** Macromolecular features of **HA(270)-FA-Pg** graft copolymers compared to those of starting **HA(270)**.

Sample	dn/dc (mL/g)	*M*_p_ (kg/mol)	*M*_w_ (kg/mol)	*M*_w_/*M*_n_	Rec. Mass ^a^ (%)	Grafting ^b^ (%)
**HA(270) ^c^**	0.150	188	274	3.89	94	0
**HA(270)-FA-Pg-10 ^d^**	0.140	278	330	2.09	80	10
**HA(270)-FA-Pg-20 ^d^**	0.140	359	387	2.21	68	20
**HA(270)-FA-Pg-40 ^d^**	0.140	347	288	2.49	21	40

^a^ Recovered Mass: the fraction of the polymeric sample eluting from the SEC columns. ^b^ The determination of the grafting degree was made by ^1^H NMR spectroscopy after hydrolysis with NaOD in D_2_O as described in ref [14]. ^c^ The values of native **HA(270)** reported in the table were obtained with 0.2 M NaCl + 0.1 M phosphate buffer pH 7.4. ^d^ The values of the graft copolymers were obtained with 0.1 M carbonate buffer pH 10.

**Table 3:** WC % (% water content by TGA), Wtot (total water by TGA), Wnf (not freezing Water by DSC), and Wf (freezing Water by DSC) % fW: percentage of freezing water. The subdivision of freezing water in free freezable water (Wff) and bound freezable water (Wbf) expressed as percentage.

Sample	30–120 °C WC (%)	Wsg (mg)	Wtot (mg)	Wf (mg)	Wff-Wbf (%)	Wnf (mg)
**HA(270)-FA-HEG-CL-10**	98	17.8	17.4 (100%)	16.2 (93%)	98-2	1.2 (7%)
**HA(270)-FA-HEG-CL-20**	97	13.8	13.4 (100%)	11.5 (87%)	82-18	1.8 (13%)
**HA(270)-FA-HEG-CL-40**	93	8.37	7.78 (100%)	6.95 (90%)	88-12	0.82 (10%)
**HA(8.7)-FA-HEG-CL-20**	97	19.4	18.8 (100%)	18.0 (95%)	97-3	0.88 (8%)

**Table 4:** The major mechanical parameters of the hydrogels obtained with **HA(270)-FA-HEG-CL**-**10**, **HA(270)-FA-HEG-CL**-**20**, **HA(270)-FA-HEG-CL**-**40**, and **HA(8.7)-FA-HEG-CL**-**20** materials.

Sample	G′ (2.5 Hz) (Pa)	G″(2.5 Hz) (Pa)	η 0.1 (Pa.s)	Shear Thinning Ratio (η0.1/η250)
**HA(270)-FA-HEG-CL-10**	356 ± 9	59 ± 24	44 ± 11	156 ± 26
**HA(270)-FA-HEG-CL-20**	887 ± 11	266 ± 18	136 ± 48	4690 ± 154
**HA(270)-FA-HEG-CL-40**	708 ± 14	147 ± 39	138 ± 52	9428 ± 269
**HA(8.7)-FA-HEG-CL-20**	13 ± 2	0.25 ± 0.09	0.47 ± 0.05	390 ± 16

## Supplementary Materials

The following supporting information can be downloaded at: https://www.mdpi.com/article/10.3390/pharmaceutics14051041/s1, ^1^H NMR spectra (D_2_O, 600 MHz) obtained with **HA(270)-FA**-**HEG-CL** and **HA(8.7)-FA**-**HEG-CL-20** cross-linked materials. Thermographs of the hydrogels obtained with **HA(270)-FA**-**HEG-CL** and **HA(8.7)-FA**-**HEG-CL-20** cross-linked materials. Viscosity values of 1% *w*/*v* solutions at different shear rate (γ) values of **HA(270)-FA**-**Pg** and **HA(8.7)-FA**-**Pg**-**20** graft copolymers.
