# Supplementary material for: Click-Chemistry Cross-Linking of Hyaluronan Graft Copolymers
Source: Pharmaceutics. 2022 May 11;14(5):1041. doi: 10.3390/pharmaceutics14051041 (PMC9146110; doi:10.3390/pharmaceutics14051041)
Supplement: Supplementary file 1 [file pharmaceutics-14-01041-s001.zip › pharmaceutics-1703697-supplementary.pdf]

Electronic Supplementary Materials for

Click-Chemistry Cross-Linking of Hyaluronan Graft Copolymers

Mario Saletti [1], Marco Paolino [1,*], Lavinia Ballerini [1], Germano Giuliani [1], Gemma Leone [1], Stefania Lamponi [1], Marco Andreassi [1], Claudia Bonechi [1], Alessandro Donati [1], Daniele Piovani [2], Alberto Giacometti Schieroni [2], Agnese Magnani [1] and Andrea Cappelli [1,*]

[1] Dipartimento di Biotecnologie, Chimica e Farmacia (Dipartimento di Eccellenza 2018–2022), Università degli Studi di Siena, Via Aldo Moro 2, 53100 Siena, Italy; mario.saletti@student.unisi.it (M.S.); lavinia.ballerini@student.unisi.it (L.B.); giuliani5@unisi.it (G.G.); gemma.leone@unisi.it (G.L.); stefania.lamponi@unisi.it (S.L.); marco.andreassi@unisi.it (M.A.); claudia.bonechi@unisi.it (C.B.); alessandro.donati@unisi.it (A.D.); agnese.magnani@unisi.it (A.M.)

[2] Istituto di Scienze e Tecnologie Chimiche "G. Natta"-SCITEC (CNR), Via A. Corti 12, 20133 Milano, Italy; piovani@ismac.cnr.it (D.P.); alberto.giacometti@scitec.cnr.it (A.G.S.)

* Correspondence: paolino3@unisi.it (M.P.); andrea.cappelli@unisi.it (A.C.); Tel.: +39-0577-234320 (A.C.)

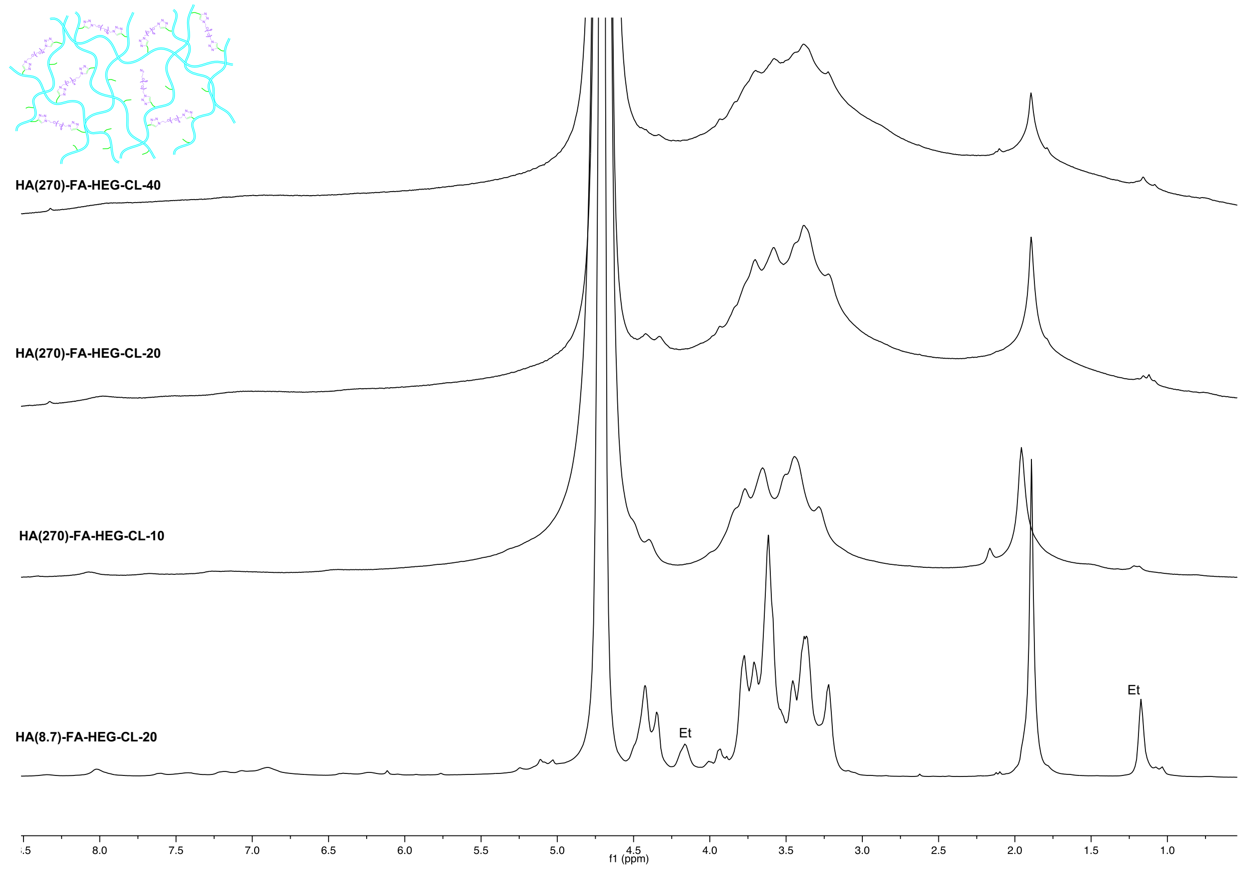

Figure S1. Comparison ^{1}H NMR spectra (D_2O, 600 MHz) obtained with the cross-linked materials **HA(8.7)-FA-HEG-CL-20**, **HA(270)-FA-HEG-CL-10**, **HA(270)-FA-HEG-CL-20**, **HA(270)-FA-HEG-CL-40**. In the

spectrum of **HA(8.7)-FA-HEG-CL-20**, “Et” labels indicate the signals of ethyl groups of the monomeric units with esterified carboxylic groups.

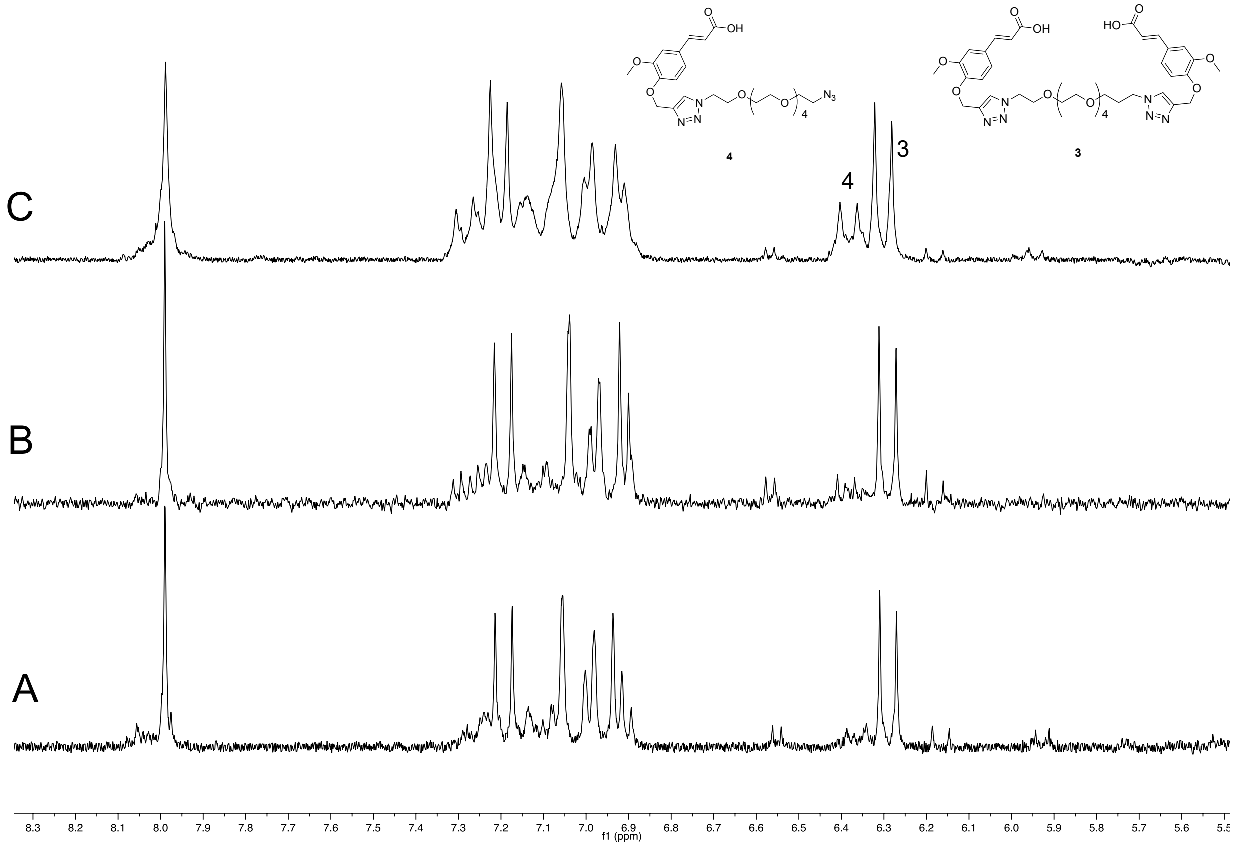

Figure S2. Comparison of the aromatic regions of ^{1}H NMR spectra (D_2O, 400 MHz) obtained with the cross-linked materials **HA(270)-FA-HEG-CL-10** (trace A), **HA(270)-FA-HEG-CL-20** (trace B), **HA(270)-FA-HEG-CL-40** (trace C) after hydrolysis with NaOD at room temperature for 10 min.

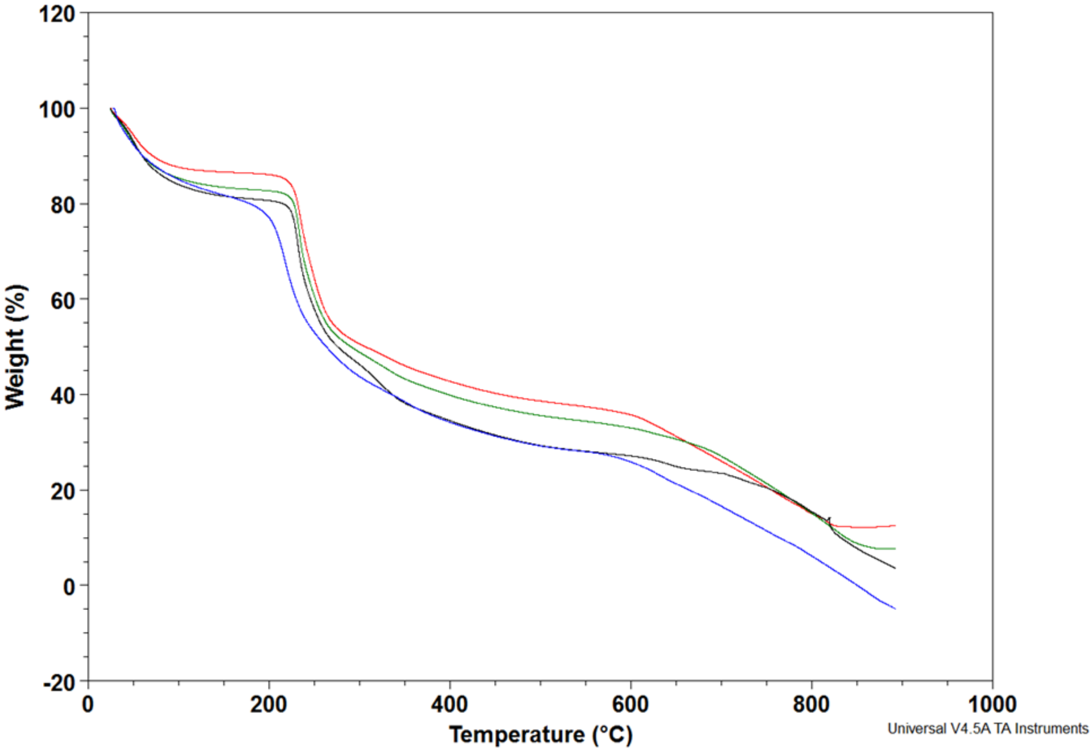

Figure S3. Thermographs of the hydrogels obtained with **HA(270)-FA-HEG-CL-20** (green), **HA(270)-FA-HEG-CL-40** (black), **HA(270)-FA-HEG-CL-10** (red), **HA(8.7)-FA-HEG-CL-20** (blue).

Table S1. Viscosity values of 1% w/v solutions at different shear rate (γ) values of **HA(270)-FA-Pg** and **HA(8.7)-FA-Pg-20** graft copolymers. Zero-shear viscosity (η0) obtained applying Cross Model.

Sample	η (γ= 0.01 s^{-1})	η (γ= 0.01 s^{-1})	η0	R^2
**HA(270)-FA-Pg-10**	4.6 ± 0.9	$7.8 \pm 0.4 \cdot 10^{-3}$	6.6 ± 0.3	1
**HA(270)-FA-Pg-20**	2.0 ± 1.0	$1.2 \pm 0.3 \cdot 10^{-2}$	2.9 ± 0.8	1
**HA(270)-FA-Pg-40**	8.8 ± 0.4	$2.3 \pm 0.4 \cdot 10^{-2}$	26.9 ± 0.8	1
**HA(8.7)-FA-Pg-20**	3.1 ± 0.2	$6.4 \pm 0.2 \cdot 10^{-4}$	1.4 ± 0.2	0.97
